# Single-Nucleus RNA Sequencing Reveals the Spatiotemporal Dynamics of Disease-Associated Microglia in Amyotrophic Lateral Sclerosis

**DOI:** 10.34133/research.0548

**Published:** 2024-12-11

**Authors:** Lu-Xi Chen, Mei-Di Zhang, Hai-Feng Xu, Hai-Qin Ye, Dian-Fu Chen, Pei-Shan Wang, Zhi-Wei Bao, Sheng-Mei Zou, Yong-Ting Lv, Zhi-Ying Wu, Hong-Fu Li

**Affiliations:** ^1^ Department of Medical Genetics and Center for Rare Diseases, Second Affiliated Hospital, Zhejiang University School of Medicine, Hangzhou, Zhejiang, China.; ^2^ Nanhu Brain-Computer Interface Institute, Hangzhou, China.; ^3^ Zhejiang Key Laboratory of Rare Diseases for Precision Medicine and Clinical Translation, Hangzhou, Zhejiang, China.; ^4^ Department of Neurology, Second Affiliated Hospital, Zhejiang University School of Medicine, Hangzhou, China.; ^5^ Collaborative Innovation Center for Diagnosis and Treatment of Infectious Diseases, The First Affiliated Hospital, Zhejiang University School of Medicine, Hangzhou, China.; ^6^ Institute of Developmental and Regenerative Biology, Zhejiang Key Laboratory of Organ Development and Regeneration, College of Life and Environmental Sciences, Hangzhou Normal University, Hangzhou, China.; ^7^ MOE Frontier Science Center for Brain Science and Brain-Machine Integration, School of Brain Science and Brain Medicine, Zhejiang University, Hangzhou, China.

## Abstract

Disease-associated microglia (DAM) are observed in neurodegenerative diseases, demyelinating disorders, and aging. However, the spatiotemporal dynamics and evolutionary trajectory of DAM during the progression of amyotrophic lateral sclerosis (ALS) remain unclear. Using a mouse model of ALS that expresses a human *SOD1* gene mutation, we found that the microglia subtype DAM begins to appear following motor neuron degeneration, primarily in the brain stem and spinal cord. Using reverse transcription quantitative polymerase chain reaction, RNAscope in situ hybridization, and flow cytometry, we found that DAM increased in number as the disease progressed, reaching their peak in the late disease stage. DAM responded to disease progression in both SOD1^G93A^ mice and sporadic ALS and *C9orf72*-mutated patients. Motor neuron loss in SOD1^G93A^ mice exhibited 2 accelerated phases: P90 to P110 (early stage) and P130 to P150 (late stage). Some markers were synchronized with the accelerated phase of motor neuron loss, suggesting that these proteins may be particularly responsive to disease progression. Through pseudotime trajectory analysis, we tracked the dynamic transition of homeostatic microglia into DAM and cluster 6 microglia. Interestingly, we used the colony-stimulating factor 1 receptor (CSF1R) inhibitor PLX5622 to deplete microglia in SOD1^G93A^ mice and observed that DAM survival is independent of CSF1R. An in vitro phagocytosis assay directly confirmed that DAM could phagocytose more beads than other microglia subtypes. These findings reveal that the induction of the DAM phenotype is a shared cross-species and cross-subtype characteristic in ALS. Inducing the DAM phenotype and enhancing its function during the early phase of disease progression, or the time window between P130 and P150 where motor neuron loss slows, could serve as a neuroprotective strategy for ALS.

## Introduction

Microglia originate from primitive macrophages in the yolk sac, which migrate into the developing central nervous system [1]. These cells have the ability to self-proliferate, maintaining their population throughout life. In their resting state, microglia are ramified cells, but they transform into activated, larger amoeboid cells with retracted processes in response to insults [2]. In this activated state, they engulf and degrade extracellular aggregates and debris and promote tissue repair [3–5]. The immune activity of microglia is regulated by specific immune inhibitory pathways that suppress undesirable inflammatory responses and prevent tissue damage commonly associated with immune activation [6]. Dysregulation of microglia is a primary mediator and hallmark of neuroinflammation in various neurological disorders, including amyotrophic lateral sclerosis (ALS), Alzheimer’s disease (AD), Parkinson’s disease, multiple sclerosis, stroke, autism, and schizophrenia [7–9].

ALS is the third most common neurodegenerative disease with a lifelong risk of 1/400 [10]. It causes devastating degeneration of motor neurons in the spinal cord and brain stem, leading to significant paralysis of voluntary muscle groups and ultimately resulting in death due to respiratory failure within 3 to 5 years after symptom onset [11]. Currently, few effective pharmacological treatments modify the disease’s relentless course. The long-approved drug riluzole extends survival by a few months [12,13], and edaravone provides limited clinically relevant benefits [14,15]. Recently, the approved combination therapy Relyvrio, consisting of sodium phenylbutyrate and taurursodiol, has been discontinued due to its failure to demonstrate benefits in a confirmatory phase 3 trial. Ninety percent of ALS cases are sporadic without a family history, while 10% are due to inherited genetic mutations. Genetic therapy is advancing rapidly, and the antisense oligonucleotide tofersen designed for *SOD1*-mutated ALS patients has been approved for marketing. However, mutations within the *SOD1* gene account for about 20% of familial ALS (fALS) cases, which translates to 1% to 2% of all ALS cases [16]. The failure of numerous clinical trials targeting motor neuron death mechanisms has led to the realization that modulating non-cell-autonomous mechanisms may represent a promising new direction [17]. To date, the majority of clinical trials for ALS patients target the non-cell-autonomous mechanism, highlighting the pivotal role of microglia in disease progression [17].

Before the advent of single-nucleus sequencing technology, a major challenge in microglia research was the reliance on only 2 markers, CD45 and CD11b, for their isolation. This led to data that reflected microglia as a homogeneous population, thereby overlooking the crucial information about the heterogeneity of microglia associated with specific pathologies. Single-cell RNA sequencing (scRNA-seq) has since identified disease-associated microglia (DAM), a specific subtype of microglia initially observed in a mouse model of AD that expresses 5 human familial AD mutations (5XFAD), which exhibit a distinct phenotype that does not align with either the M1 or M2 state [18]. The study also detected DAM in immune cells from spinal cords of an ALS mouse model [18], further validated by scRNA-seq of the whole spinal cord in the same ALS mouse model [19]. However, there is a lack of systematic study demonstrating the spatiotemporal dynamics of DAM in ALS progression. Furthermore, the molecular changes in the evolutionary trajectory of DAM during ALS progression remain unknown. In this study, we combine high-throughput sequencing with experimental approaches to explore the spatiotemporal changes in DAM during the progression of ALS. Additionally, we identified a critical window for therapeutic intervention between P110 and P130, where motor neuron loss slows, providing an optimal period for therapeutic intervention.

## Results

### The single-cell transcriptome in a mouse model of fALS is elucidated

**Fig. 1.**
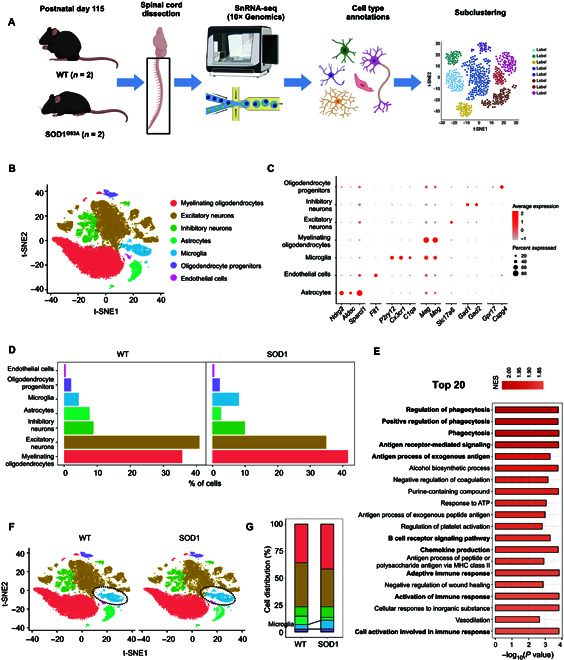
Single-nucleus RNA sequencing (snRNA-seq) of spinal cords in the SOD1^G93A^ mouse model of amyotrophic lateral sclerosis (ALS). (A) Schematic summarizing design: whole spinal cords from postnatal day 115 SOD1^G93A^ and wild-type (WT) littermate male mice (*n* = 2/group) were prepared and sequenced. (B) t-distributed stochastic neighbor embedding (t-SNE) plot of cell types in 2 groups. (C) Dot plot of canonical marker genes [19] for identified cell types. (D) Bar plots showing the proportion of each cell type. (E) Bar plot displaying the top 20 Gene Ontology (GO) pathways analyzed by gene set enrichment analysis (GSEA) enriched in differentially expressed genes (DEGs) between the SOD1^G93A^ and WT groups. (F) t-SNE plots of cells in WT and SOD1^G93A^ mice. (G) Proportion of each cell type separated by 2 groups. The number of microglia in the SOD1^G93A^ group is much higher than that in the WT group. NES, normalized enrichment score; ATP, adenosine triphosphate; MHC, major histocompatibility complex.

Single-nucleus RNA sequencing (snRNA-seq) was performed using 10× Genomics technology on cells from spinal cords derived from mice harboring human mutations in the *SOD1* gene. The SOD1^G93A^ mice represent the first established and widely utilized model of fALS, originally developed in 1994 (Fig. 1A) [20]. We analyzed a total of 49,910 single nuclei isolated from 4 adult male mice (2 biological replicates for SOD1^G93A^ and littermate controls), aged approximately 4 months (P115), utilized the Seurat package for unsupervised clustering [21,22]. After quality control (QC), an average of 11,794 cells (88.3% passed QC) per sample from the wild-type (WT) group and 10,548 cells (90.9% passed QC) per sample from the SOD1^G93A^ group were included in subsequent analyses (Tables S1 and S2). The sequencing depths were approximately ~20,000 reads per cell, and the median count of unique molecular identifiers before and after QC is depicted in Fig. S1.

Seven cell types were identified and visualized by t-distributed stochastic neighbor embedding plotting (Fig. 1B). The identities of the cell types were determined using differential expression analysis of genes and canonical marker genes specific to each cell type (Fig. 1C and Fig. S2A and B) [19,23]. All cell populations were identified in both groups, with neurons accounting for nearly half (Fig. 1D and Fig. S2C and D). Differential expression analysis of neurons, including excitatory neurons and inhibitory neurons, is shown in Table S3. Differential expression analysis in the whole spinal cord cells between SOD1^G93A^ and WT mice yielded 95 differentially expressed genes (DEGs) (Table S4), a number consistent with previous datasets [19]. In addition, both the gene and pathway levels, as the top 20 gene set enrichment analysis (GSEA) Gene Ontology (GO) pathways, indicated significant differences in the functions of phagocytosis and immune response between SOD1^G93A^ mice and WT mice (Fig. 1E and Table S5). It is well-known that microglia are the primary resident immune cells in the central nervous system, involved in physiological processes such as phagocytosis, debris elimination, immune cell recruitment, and neurogenesis [24]. Notably, an increase (4% to 8%) in microglia population was observed in SOD1^G93A^ mice compared to that in controls (Fig. 1F and G), which sparked our curiosity to investigate how microglia contribute to ALS progression.

### Microglia subsets: DAM and cluster 6 microglia show a dramatic increase in SOD1^G93A^ mice

**Fig. 2.**
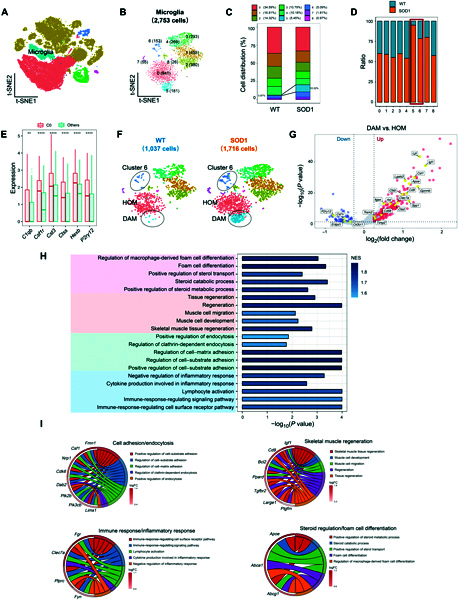
Disease-associated microglia (DAM) show a dramatic increase in SOD1^G93A^ mice. (A) t-SNE plot highlights the microglia in color blue. (B) t-SNE plot showing 9 subclusters of microglia population with “resolution = 0.8”. (C) Stacked bar graph showing the average percentages of cells in each cluster of microglia in WT and SOD1^G93A^ mice. (D) Comparison of the proportions of microglia subpopulations in WT and SOD1^G93A^ mice. The number of cells in the C5 and C6 of the SOD1^G93A^ group is higher than that in the WT group, especially in cluster 5. (E) Bar plots showing the expression level of 6 homeostatic microglia (HOM) markers in C0 compared to those in all other microglia subclusters. **P value < 0.01, ****P value < 0.0001, C0 vs. others. (F) t-SNE plot showing subcluster annotations of microglia. The numbers of microglia in WT and SOD1^G93A^ are in parentheses. (G) Volcano plot showing the DEGs between DAM and HOM. Blue dots represent down-regulated DEGs, and red dots represent up-regulated DEGs. Genes related to DAM and HOM signature are labeled yellow. (H) GO enrichment analysis was performed by GSEA on genes ranked by log_2_FC between DAM and HOM. Bar plot showing 4 major pathway categories: “steroid regulation/foam cell differentiation” colored pink, “skeletal muscle regeneration” colored orange, “cell adhesion/endocytosis” colored green, and “immune/inflammatory response” colored blue. (I) The relationships between the activated GO terms in (H) and the core genes are depicted. Circos plots showing the core genes related to “cell adhesion/endocytosis”, “immune/inflammatory response”, “skeletal muscle regeneration”, and “steroid regulation/foam cell differentiation” functions. FC, fold change.

To explore the heterogeneity of microglia, unsupervised clustering analysis was performed on microglia characterized by high expressions of *P2ry12*, *Cx3cr1*, and *C1qa* containing 2,753 cells (Fig. 2A). In the microglia of both WT and SOD1^G93A^ mice, 9 distinct subclusters (clusters 0 to 8) of microglia were identified (Fig. 2B and Table S6). Consistent with previous findings [18], cluster 5 was identified as DAM based on significantly high UCell scores and expression levels of *Apoe*, *Cd9*, *Trem2*, *Ctsd*, *Csf1*, and *Ctsb* (Fig. S3A and B). DAM showed a robust increase from nearly absent in WT mice to 10.01% in SOD1^G93A^ mice (Fig. 2C). Interestingly, we observed a remarkable rise in cluster 6 as well within the ALS model (Fig. 2D). Cluster 0 accounted for almost 35% of the total number of microglia and was identified as homeostatic microglia (HOM), characterized by significantly higher expression of *Csf1r*, *Cst3*, *Ctss*, *Hexb*, and *P2ry12* (Fig. 2E and F). Furthermore, GO enrichment analysis of DEGs between HOM and the other microglia subclusters revealed extensive inhibition (Table S7) [19,25]. Consistent with prior research, DAM significantly down-regulated the gene expression levels of HOM markers, including the purinergic receptors *P2ry12*, *Cx3cr1*, and *Csf1r* [18]. Compared to HOM, DAM showed up-regulation of numerous genes, including well-known AD risk factors such as *Apoe*, *Ctsd*, *Lpl*, *Trem2*, and other genes such as *Igf1*, *Csf1*, *Lgals3*, *Cd9*, *Ctsb*, *Gpnmb*, *Itgax*, *Axl*, *Ctsb*, *Cd68*, and *Timp2* (Fig. 2G and Table S8). GSEA GO pathway analysis identified the pathways and corresponding core DEGs associated with “cell adhesion/endocytosis”, “immune/inflammatory response”, “skeletal muscle regeneration”, and “steroid regulation/foam cell differentiation” in DAM (Fig. 2H and Table S7). Using chord plots, we further displayed the relationship between significantly changed genes and corresponding GO terms (Fig. 2I).

**Fig. 3.**
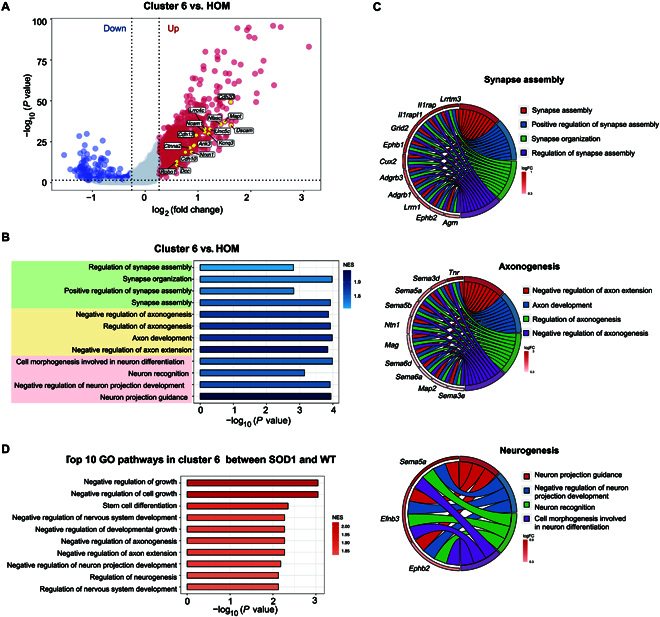
Cluster 6 microglia show a dramatic increase in SOD1^G93A^ mice. (A) Volcano plot showing the DEGs between cluster 6 microglia and HOM. Blue dots represent down-regulated DEGs, and red dots represent up-regulated DEGs. (B) GO enrichment analysis was performed on genes ranked by log_2_FC between cluster 6 microglia and HOM. Bar plot showing 3 major pathway categories: “synapse assembly”, “axonogenesis”, and “neurogenesis”. (C) The relationship between the activated GO terms in (B) and the involved genes is depicted. Circos plots show the core genes related to the pathways. (D) Bar plot displaying the top 10 GO enrichment pathways in cluster 6 between SOD1^G93A^ mice and WT mice.

Compared to HOM, the cluster 6 microglia exhibited 981 up- and 506 down-regulated DEGs (Fig. 3A and Table S9). Notably, these DEGs were associated with the “synapse assembly” pathway, the “axonogenesis” pathway, and the “neurogenesis” pathway (Fig. 3B and C). In addition, the DEGs in cluster 6 microglia between SOD1^G93A^ and WT mice further confirmed that the top 10 GO pathways mentioned above were mainly evoked (Fig. 3D). To validate the expansion of cluster 6 microglia, we performed reverse transcription quantitative polymerase chain reaction (RT-qPCR) on spinal cords from SOD1^G93A^ and WT littermates. *Tmod2* expression was significantly increased in SOD1^G93A^ mice at both postnatal days 110 and 130, while *Trappc9* showed increased expression at day 130. Both genes were among the top 10 up-regulated DEGs in cluster 6, thus supporting the expansion of cluster 6 microglia in SOD1^G93A^ mice (Fig. S4 and Table S10).

In the progression of neurodegenerative diseases, HOM and DAM have received more attention; besides that, the inflammatory response has also been described [26,27]. We asked whether cluster 6 microglia belong to this inflammatory state. Cluster 6 microglia showed a low gene expression level associated with the immune response to interferon/lipopolysaccharide (Fig. S5A and B). We subsequently evaluated the correlation between cluster 6 microglia and different phases of the microglial cell cycle by using the gene sets of different phases, including the synthesis, gap 2, mitosis, and gap 1 phases, based on a previous study describing adult microglia proliferation [28]. Cluster 6 microglia exhibited an expression pattern inconsistent with the cell cycle (Fig. S5C). Collectively, there was a significant increase in both DAM and cluster 6 microglia numbers in SOD1^G93A^ mice compared to WT mice, which indicated that these 2 particular types of microglia are essential in disease progression in SOD1^G93A^ mice.

### DAM evolve from HOM and are conserved from mouse to human neuropathology in ALS

**Fig. 4.**
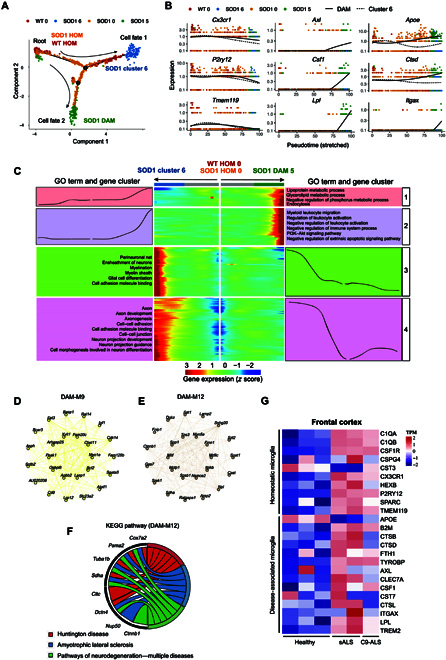
DAM evolve from homeostatic microglia and are conserved from mouse to human neuropathology in ALS. (A) Pseudotime analysis of the single-cell trajectories of HOM, DAM, and cluster 6 microglia using Monocle2. Two major branches were identified; one led to cluster 6 cells in SOD1^G93A^ from HOM (cell fate 1), and the other led to DAM in SOD1^G93A^ (Cell fate 2). (B) Representative genes related to HOM and DAM signatures were selected to show their expression trends before and after cell state branching. (C) Gene signatures during microglia state switch. The expression level of DEGs (rows) are shown using a heatmap with microglia (columns) in pseudotime from the root to cell fate 1 or cell fate 2. Gene expression trends in each group (middle). GO terms associated with DEGs in the 4 kinetic clusters (left/right). (D and E) Weighted gene coexpression network analysis (WGCNA) was performed in DAM, and identified 2 gene modules were enriched. Network plots of the top 25 genes with the highest intramodular connectivity in yellow and camel modules. (F) Kyoto Encyclopedia of Genes and Genomes (KEGG) pathways enriched by DAM-M12. The Circos plot shows genes related to the KEGG pathways of “Huntington disease”, “amyotrophic lateral sclerosis”, and “neurodegeneration—multiple diseases”. (G) Heatmap showing the markers of DAM and HOM of the frontal cortex of healthy controls, sporadic ALS, and *C9orf72*-mutated ALS (GSE67196). TPM, transcripts per million; sALS, sporadic ALS; C9-ALS, *C9orf72*-mutated ALS.

Both DAM and cluster 6 microglia, 2 distinct subtypes of microglia, exhibited apparent reactivity during the progression of the disease. Therefore, we performed pseudotime transformation trajectory analysis to computationally evaluate whether cluster 6 microglia could potentially represent an intermediate state in the transition toward a DAM state [29–31]. We found that both cluster 6 microglia and DAM displayed a gradual transition from resting microglia along separate trajectories (cell fates 1 and 2) (Fig. 4A). These results suggest that the polarization of microglia into either DAM or cluster 6 microglia originated from HOM in response to different stimuli during disease progression. The HOM signature (*Cx3cr1*, *P2ry12*, and *Tmem119*) was decreased in both DAM (cell fate 2) and cluster 6 microglia (cell fate 1) (Fig. 4B). It was revealed that genes such as *Axl*, *Csf1*, *Lpl*, and *Itgax* were barely expressed in HOM and highly expressed in DAM during disease progression (Fig. 4B). Genes such as *Apoe* and *Ctsd* showed specific increases specifically in DAM but were unaffected in cluster 6 microglia (Fig. 4B). Then, we employed branched expression analysis modeling (BEAM) to analyze and visualize the alterations in gene expression across these 3 subclusters of microglia. Notably, we observed a distinct shift in gene expression during the transition from HOM to DAM (gene clusters 1 and 2) (Fig. 4C and Table S11). The DEGs associated with GO terms between HOM and DAM were consistent with our previous findings (Figs. 2I and 4A), indicating DAM exhibited a unique activation pattern. Interestingly, despite the distinct gene expression differences between cluster 6 microglia and HOM, pathway enrichment analysis of cluster 6 revealed a significant inhibition of GO terms (Table S7), which may explain the closer relationship observed between cluster 6 microglia and HOM in Monocle analysis (Fig. 4A).

To further investigate the potential role of DAM, we conducted weighted gene coexpression network analysis (WGCNA) for DAM and identified 2 gene modules that were significantly enriched in DAM (M9 and M12) (Fig. 4D and E and Fig. S6). Interestingly, the genes in the DAM-M12 module were enriched in Kyoto Encyclopedia of Genes and Genomes disease-related pathways, including Huntington’s disease, ALS, and pathways of neurodegeneration—multiple diseases (Fig. 4F).

Importantly, we aimed to determine whether the phenomenon of DAM observed in the ALS mouse model could also be extended to patients. Therefore, we reanalyzed transcriptomic changes in the frontal cortex of healthy controls, sporadic ALS (sALS), and *c9orf72*-mutated ALS (C9-ALS) patients using dataset GSE67196 [32]. We found that DAM signatures were up-regulated in sALS and C9-ALS compared to those in the healthy control (Fig. 4G). These results suggest that DAM actively participate in the disease progression and are conserved from mouse to human neuropathology in ALS.

### DAM appear in the spinal cord and brain stem after disease onset and increase during disease progression in SOD1^G93A^ mice

**Fig. 5.**
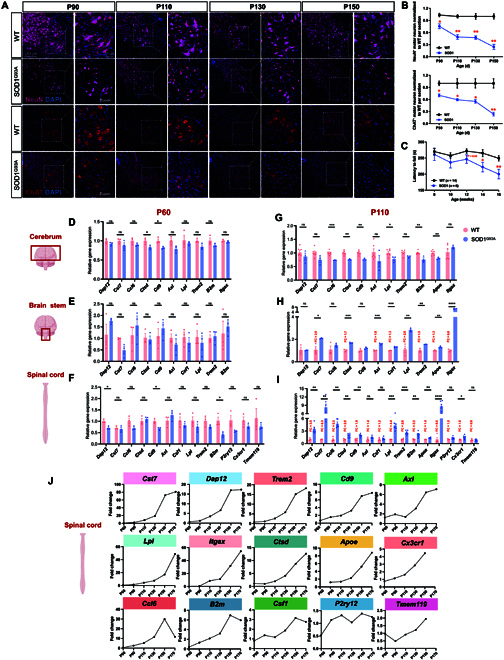
DAM appear in the spinal cord and brain stem after motor neuron death in SOD1^G93A^ mice. (A and B) Representative microscopic images and quantification of NeuN^+^ and ChAT^+^ motor neurons at the ventral horn of the lumbar spinal cord in SOD1^G93A^ and WT mice at different time points stained for NeuN. Nuclei are labeled with 4′,6-diamidino-2-phenylindole (DAPI). Scale bar: 20 μm. (C) Weekly rotarod performances of WT (*n* = 14) and SOD1 (*n* = 6) mice. Mean holding times on the rotating rod at indicated weeks were plotted. *P < 0.05 and **P < 0.01, Student *t* tests. Data are mean ± standard error of the mean (SEM). (D to I) Quantitative reverse transcription quantitative polymerase chain reaction (RT-qPCR) analysis of DAM signatures in the cerebrum, brain stem, and spinal cord at P60 and P110. Each result was normalized to *Gapdh*. Error bars denote SEM (*n* = 3 to 6). *P < 0.05, **P < 0.01, ***P < 0.001, and ****P < 0.0001, Student *t* tests. (J) A line chart showing the average fold change of the temporal evolution of each DAM marker. NeuN, neuronal nuclei; ChAT, choline acetyltransferase; ns, not significant.

ALS is a progressive disease with a gradual increase in motor neuron death and loss of motor function [16]. Identifying the dynamic changes in DAM signature expression throughout the progression of the disease could shed light on the molecular mechanisms of DAM regulation and offer potential novel therapeutic targets. First, we assessed motor neuron count at the ventral horn of the lumbar spinal cord and evaluated motor function via the rotarod test along the course of the disease. Our findings revealed significant motor neuron loss in our SOD1^G93A^ mice at 90 d, although the motor function was preserved (Fig. 5A to C). Interestingly, the death of motor neurons exhibited an accelerated pattern during 2 distinct phases: from 90 to 110 d and from 130 to 150 d. However, this decline was not synchronized with the deterioration of motor function (Fig. 5A to C). To interrogate the dynamics of DAM throughout disease progression and across various regions, we employed RT-qPCR to examine DAM and HOM marker expression levels. At P60 (pre-onset), no marked changes in DAM expression were observed in the cerebrum, brain stem, or spinal cord tissues compared to WT mice (Fig. 5D to F). By P110, substantial DAM expression changes were noted in the brain stem and spinal cord but not in the cerebrum, indicating primary DAM activation sites (Fig. 5G to I). Subsequently, the DAM markers in spinal cord tissue were assessed at 90, 130, 150, and 170 d (Fig. S7A to D). At disease onset (90 d), a slight increase in fold change is observed for a few markers (*Cst7*, *Apoe*, and *B2m*) of DAM in the spinal cord of SOD1^G93A^ mice (Fig. S7A). A line chart illustrated the temporal evolution of each marker in the spinal cord (Fig. 5J), showing a gradual increase in 5 DAM markers (*Cst7*, *Dap12*, *Trem2*, *Cd9*, and *Axl*) after 90 d, with a significant acceleration between 130 and 150 d (Fig. 5J and Fig. S7B and C). Four DAM markers (*Lpl*, *Itgax*, *Ctsd*, and *Apoe*) and one HOM marker (*Cx3cr1*) increased gradually from 90 to 130 d, followed by a pronounced surge from 130 to 170 d (Fig. 5J and Fig. S7). The expression levels of 3 DAM markers (*Ccl6*, *B2m*, and *Csf1*) increased progressively until 150 d, followed by a decline from 150 to 170 d (Fig. 5J and Fig. S7).

**Fig. 6.**
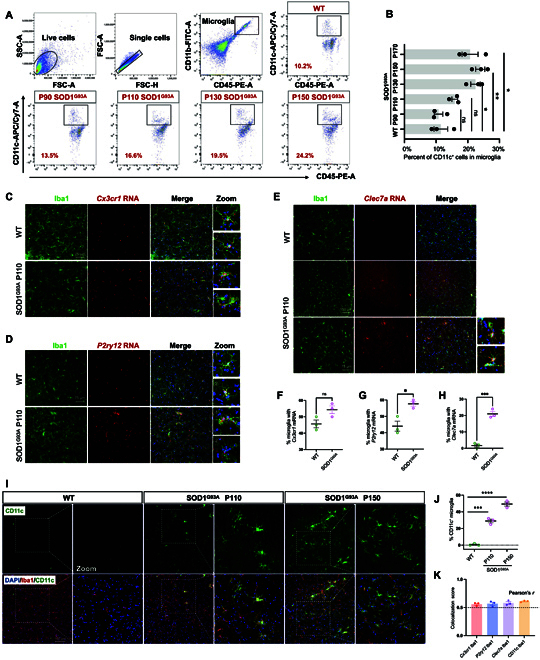
DAM exhibit an increase during disease progression in SOD1^G93A^. (A) Flow cytometry analysis of CD11c^+^CD11b^+^CD45^+^ DAM in the spinal cord (per 10^4^ total microglia) at 90, 110, 130, 150, and 170 d in SOD1^G93A^ and WT mice. (B) Statistics of the percent of CD11c^+^ DAM at different time points. *P < 0.05 and **P < 0.01, Student *t* tests. (C to E) Fresh-frozen spinal cords from 110-d WT and SOD1^G93A^ littermates were sectioned and prepared using RNAscope with the probes (*Cx3cr1*, *P2ry12*, and *Clec7a*). Scale bar: 75 μm. (F to H) Quantification of (C to E) the percent of messenger RNA (mRNA)-probe-positive microglia analyzed per image. *n* = 3 animals per group; *n* = 3 to 4 images from the ventral horn (1 image per section). *P < 0.05; ***P < 0.001; ns, not significant; Student *t* tests. Data are represented as mean ± SEM. (I) Representative images from the lumbar spinal cord in SOD1^G93A^ mice and their littermates at 110 and 150 d, stained for a DAM marker gene (CD11c, green), ionized calcium-binding adapter molecule 1 (Iba1; microglia, red), and DAPI (cell nuclei, blue). Scale bar: 100 μm. (J) Quantification of percent of CD11c^+^ microglia analyzed per image. *n* = 3 animals per group; *n* = 3 to 4 images from ventral horn (1 image per section). ***P < 0.001, ****P < 0.0001, Student *t* tests. (K) Colocalization analysis of mRNA (*Cx3cr1*, *P2ry12*, and *Clec7a*) and protein (CD11c) with microglia (Iba1) using Pearson’s correlation coefficient (Pearson’s *r*). A Pearson’s *r* above 0.5 typically indicates a significant level of colocalization. SSC-A, side scatter area; FSC-A, forward scatter area; FSC-H, forward scatter height; PE-A, phycoerythrin-A; APC, allophycocyanin; Cy7-A, cyanine 7-A.

We also tracked temporal changes in the CD11c^+^CD11b^+^CD45^+^ (DAM) population in the spinal cord using flow cytometry from 90 to 170 d in SOD1^G93A^ mice, thereby spanning stages from pre-onset to terminal (Fig. 6A). In accordance with RT-qPCR findings, no important differences in DAM population were observed at 90 d between SOD1^G93A^ and WT mice. The numbers of infiltrating DAM significantly increased from 110 d onward, peaked at 150 d, and stabilized by 170 d (Fig. 6B). Growing studies pay more attention on sex-specific differences in microglia in neurodegenerative diseases [33–35]. Then, we conducted RT-qPCR on spinal cords from both genders at postnatal day 110 and found no marked differences in DAM signatures (Fig. S8A). Additionally, we also performed flow cytometry to directly compare the counts of CD11c^+^ microglia between male and female SOD1^G93A^ mice at postnatal day 130 and found no differences in DAM numbers during disease progression (Fig. S8B). Therefore, we propose that there are no substantial sex-related disparities in DAM signatures or DAM counts during disease progression.

To further validate the snRNA-seq findings, we performed RNAscope in situ hybridization (ISH) for *Cx3cr1*, *P2ry12*, and *Clec7a*, coupled with immunofluorescent detection of microglia (ionized calcium-binding adapter molecule 1 [Iba1]), neurons (neuronal nuclei [NeuN]), and astrocytes (glial fibrillary acidic protein [GFAP]) in the ventral horn of the lumbar spinal cord of P110 SOD1^G93A^ mice. We found that *Cx3cr1*, *P2ry12*, and *Clec7a* were exclusively expressed in microglia, not in neurons or astrocytes, as indicated by Pearson’s correlation coefficient (Fig. 6K and Fig. S9). Consistent with RT-qPCR results, the microglial *Cx3cr1* messenger RNA (mRNA) showed up-regulation with no important difference, while microglial *P2ry12* mRNA was significantly elevated (Fig. 6C, D, F, and G). Notably, microglial *Clec7a* mRNA was nearly absent in WT mice but showed robust elevation in SOD1^G93A^ mice (Fig. 6E and H). Subsequently, we performed immunostaining of the lumbar spinal cord for Iba1 together with CD11c and observed minimal colocalization in WT mice (Fig. 6I and J). In contrast, a substantial population of microglia coexpressing 2 markers was identified at about 30% in P110 and 50% in P150 (Fig. 6I and J). Using a variety of experimental methods, we have demonstrated a progressive increase in DAM population at both mRNA and protein levels after motor neuron death, primarily in the spinal cord and brain stem.

### DAM are independent of CSF1R for survival and their phagocytic capacity is enhanced

**Fig. 7.**
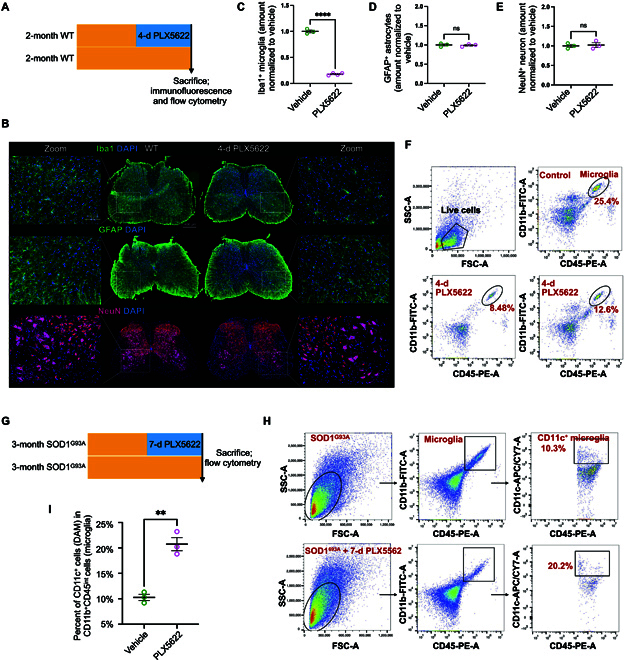
DAM are independent of colony-stimulating factor 1 receptor (CSF1R) for survival. (A) Schematic summarizing design: 2-month WT mice were fed with or without PLX5622 for 4 d and subsequently sacrificed for immunofluorescence and flowcytometry. (B) Representative microscopic images of lumbar spinal cord stained with Iba1 for microglia, glial fibrillary acidic protein (GFAP) for astrocytes, and NeuN for neurons in WT mice with or without PLX5622. Scale bar: 100 μm. (C to E) Quantitative percentage of microglia, astrocytes, and neurons between vehicle and PLX5622 in WT mice. *n* = 3 animals per group; *n* = 3 to 4 images from the ventral horn (1 image per section). (F) Flow cytometry analysis showing the percentage of microglia (CD11b^+^CD45^+^). The top right panel shows microglia from control mice, while each scatter plot in the bottom row corresponds to an individual mouse treated with PLX5622 for 4 d. (G) Schematic summarizing design: 3-month SOD1^G93A^ mice were fed with or without PLX5622 for 7 d and subsequently sacrificed for flowcytometry. (H) Flow cytometry analysis of CD11b^+^CD45^+^ microglia and CD11c^+^CD11b^+^CD45^+^ DAM between vehicle and PLX5622 in SOD1^G93A^ mice. (I) Quantitative percent of DAM of microglia after using PLX5622. **P < 0.01, ****P < 0.0001, Student *t* tests.

The survival, maintenance, and proliferation of microglia are critically dependent on colony-stimulating factor 1 receptor (CSF1R). Genetic ablation or pharmacological inhibition of CSF1R causes microglia depletion in mice [36]. To investigate whether DAM are dependent on CSF1R, we established a microglial depletion model using PLX5622, a novel inhibitor of CSF1R that effectively eliminates microglia with a higher selectivity and brain penetrance (Fig. 7A) [37,38]. Following a 4-d administration on 2-month WT mice, immunofluorescence revealed microglia depletion by more than 80% (Fig. 7B and C), while astrocytes and neurons remained relatively unaffected (Fig. 7B, D, and E). In accordance with this, fluorescence-activated cell sorting analysis demonstrated a marked decrease in the percentage of CD11b^+^CD45^medium^ microglia in WT mice (Fig. 7F). Subsequent administration of PLX5622 for 7 d in 3-month-old SOD1^G93A^ mice resulted in a substantial down-regulation of microglia percentage from 6.67% to 1.68%, as determined by fluorescence-activated cell sorting analysis (Fig. 7G). Conversely, the percentage of CD11c^+^ microglia was significantly up-regulated from 10.3% to 20.2% (Fig. 7H and I). These findings suggest that the survival of DAM is partially independent of CSF1R.

**Fig. 8.**
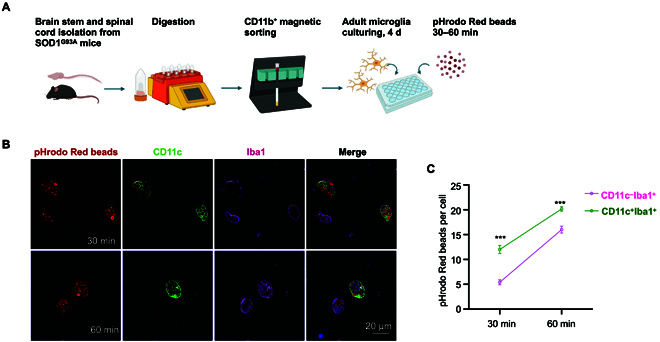
The phagocytic capacity of DAM is enhanced. (A) Schematic summarizing design: the brain stem and spinal cord were isolated from SOD1^G93A^ mice and digested into single cells using a gentleMACS Dissociator. Adult microglia were sorted by CD11b^+^ magnetic beads and cultured in plates for 4 d, and a phagocytosis assay was subsequently performed. (B) Confocal images of pHrodo Red beads in CD11c^+^Iba1^+^ (DAM) and CD11c^−^Iba1^+^ (the other microglia) at 30 and 60 min. Scale bar: 20 μm. (C) Quantification of pHrodo Red beads per microglia (CD11c^+^Iba1^+^ vs. CD11c^−^Iba1^+^) at 30 and 60 min, respectively. An average of 20 microglia were counted per well (*n* = 3). ***P < 0.001, Student *t* tests.

Our previous transcriptional analysis gives a hint that endocytosis function was enriched in DAM, but direct experimental evidence was lacking. Therefore, we cultured adult microglia from the spinal cord and brain stem of 4-month-old SOD1^G93A^ mice and exposed them to zymosan particles derivatized with pHrodo, a fluorescent indicator of cellular uptake to acid compartments and lysosomes (Fig. 8A). Our in vitro phagocytosis assay demonstrated that CD11c^+^Iba1^+^ microglia (DAM) phagocytosed more pHrodo Red beads than CD11c^−^Iba1^+^ at both 30 and 60 min (Fig. 8B and C), indicating an enhanced phagocytic capacity in DAM.

## Discussion

In this study, we demonstrated the presence of DAM using snRNA-seq from the spinal cords of SOD1^G93A^ mice. Our findings align with previous studies that identified DAM in an ALS mouse model [18,19]. Importantly, we extend these observations by showing that the DAM signature is also elevated in a public dataset of the frontal cortex of sALS and C9-ALS patients. A recent study presented a single-nucleus transcriptomic and epigenomic atlas of the frontal cortex in ALS cases, including samples from the orbitofrontal cortex of C9-ALS (*n* = 10), sALS (*n* = 8), and nonneurological controls (*n* = 6), highlighting a shift in DAM from HOM in ALS [39]. Moreover, a previous study showed increased DAM signatures in the spinal cord of sALS patients by RT-qPCR [40], suggesting that the DAM phenotype is conserved across species and ALS subtypes. DAM have been primarily detected in an AD mouse model (5XFAD), as well as aged mice (20-month-old) [18], with further observations in tauopathy models (Tau P301L and Tau P301S) [41,42] and multiple sclerosis [43]. These findings lead us to propose that the induction of the DAM phenotype is a common feature across various neurodegenerative diseases.

Our study goes beyond simply identifying DAM, as we systematically decipher the spatiotemporal dynamics of DAM throughout ALS disease progression (Table S12). We examined 6 time points and different regions (cerebrum, spinal cord, and brain stem) to track the evolution of DAM and their transcriptional changes in relation to motor neuron loss. Notably, DAM first appear after postnatal day 90, a point at which motor neurons have already undergone a 27% loss, while microgliosis is evident as early as day 30, based on the spatial transcriptomics of SOD1^G93A^ mouse spinal cords [44]. DAM are restricted to the spinal cord and brain stem—regions of motor neuron degeneration—suggesting that their activation is likely triggered by danger molecules from dying or deceased motor neurons. In addition to dying neural cells, other studies propose that myelin debris, lipid degradation products, and extracellular protein aggregates, common in neurodegenerative diseases, may serve as “danger signals” that activate DAM [45].

We observed 2 accelerated phases of motor neuron loss through NeuN and choline acetyltransferase staining: the early stage (P90 to P110) and the late stage (P130 to P150). This aligns with clinical findings in ALS patients, who experience the most rapid rates of decline during early and late phases [46]. Certain DAM marker levels showed a synchronized increase during 2 accelerated phases of motor neuron loss, indicating that these genes are more sensitive to motor neuron death. Axl, one of the TAM receptors (Tyro3, Axl, and Mer), regulates the phagocytic clearance of apoptotic neurons, plaques, and debris during neurodegeneration [47]. *Trem2* has been identified as a risk factor for AD, while the Trem2 protein plays a protective role against the toxic TDP43 protein in neuronal cells [48]. The substantial increase in the expression of *Cst7*, *Lpl*, and *Itgax*—by more than 50-fold—during disease progression suggests their critical role in activating DAM in response to neurodegeneration.

The study by Keren-Shaul et al. [18] identified the origin of DAM through scRNA-seq and specific gene expression markers but did not explore the evolutionary trajectory. Through pseudotime trajectory analysis, we tracked the dynamic transition of HOM into DAM and cluster 6 microglia and provided a detailed transcriptional analysis of how DAM evolve from HOM. Compared to HOM, DAM exhibited down-regulated expression of the checkpoints *Cx3cr1* and *P2ry12* (Table S8). Surprisingly, in contrast to DAM in AD mouse models, DAM in ALS models significantly up-regulated inhibitory checkpoints (*Cx3cr1* and *P2ry12*), as validated by ISH (Fig. 6C and D). Previous studies have shown that a specific single-nucleotide polymorphism in the *Cx3cr1* gene is associated with a shorter survival time and faster disease progression in sALS [49]. Up-regulated genes in DAM point toward GO pathways like “endocytosis” and “the inflammatory response”, which are well-known functions of DAM. Interestingly, the gene activation in DAM reveals unexpected biological processes such as “skeletal muscle regeneration” and “steroid regulation”. It suggests that DAM not only may react to neuronal damage but might also play a role in muscle regeneration in the context of ALS, where muscle atrophy occurs.

The CSF1R inhibitor PLX5622 was employed to eliminate the majority of microglia, except for resilient DAM in SOD1^G93A^ mice. While previous studies have demonstrated that DAM can survive despite CSF1R inhibition in AD mouse models [50,51], there are notable differences between these disease models. ALS primarily affects motor neurons, with damage localized to the spinal cord and brain stem, whereas AD pathology is centered around amyloid plaques and tau tangles in the hippocampus and cortex. Consequently, the inflammatory activation of microglia in ALS differs from that in AD. The survival of DAM in ALS, despite CSF1R inhibition, may be due to decreased expression of *Csf1r* (Table S8) or rely on alternative survival pathways distinct from those in AD, potentially driven by the unique microenvironment of ALS. This phenomenon may provide insight into the mechanisms by which the CSF1R inhibitor could benefit ALS patients in clinical trials [52], as these drugs may selectively deplete overactive microglia while preserving phagocytic DAM.

Previous studies have suggested that DAM exhibit stronger phagocytic abilities compared to HOM and other microglial subtypes, as indicated by increased transcriptional markers associated with phagocytosis [45]. In our study, in vitro phagocytosis assays revealed that DAM engulfed significantly more beads than other microglial subtypes. This direct visualization of enhanced phagocytic activity in DAM further supports the hypothesis, providing clearer evidence of their role in removing cellular debris during ALS progression.

There are several limitations to this study. First, this study includes a limited number of biological replicates (2 SOD1^G93A^ and 2 WT control mice). Future studies should incorporate larger cohorts to account for biological variability. Second, due to limited access to postmortem spinal cords from ALS patients, we reanalyzed public transcriptomic datasets to provide evidence for the presence of DAM in sALS and C9-ALS patients. Third, we observed 2 accelerated phases of motor neuron loss in SOD1^G93A^ mice (P90 to P110 and P130 to P150). However, the exact triggers of DAM activation and acquiring a distinct transcriptional and functional signature are not fully explored. Understanding the causative factors leading to microglial transformation into DAM could offer deeper insights and guide therapeutic interventions. Fourth, CD11c was one of the most up-regulated markers (showing an increase from undetectable levels to a 60-fold increase during late disease progression); thus, we used CD11c^+^ microglia to represent DAM in flow cytometry. CD11c is predominantly expressed in microglia that show phagocytic properties. However, it is also expressed in other myeloid cells, such as dendritic cells and subsets of macrophages, which may lead to potential confounding effects.

In conclusion, current treatments for ALS are limited, and microglia, as key components of the brain’s immune system, represent promising therapeutic targets. DAM, as a distinct microglial phenotype, may help restrict disease progression in ALS. Thus, a future neuroprotective strategy could involve identifying cytokines or small-molecule drugs that induce the DAM phenotype and enhance its function during the early phase of disease progression or even before disease onset.

## Materials and Methods

### Mice

ALS model transgenic SOD1^G93A^ mice were obtained from Baige Laboratory (Zhejiang University) and maintained in full-barrier facilities. Note that all mice employed in this study were generated on a C57BL/6J background. All mice were maintained under pathogen-free conditions and housed in a temperature-controlled (19 to 23 °C) and humidity-regulated (30% to 70% relative humidity) environment with a 12-h light/dark cycle. All animal studies and experimental procedures were approved by the Animal Care and Use Committee of the animal facility at Hangzhou Normal University. Mice were randomly assigned to either vehicle or PLX5622 treatment through a lottery drawing box to ensure unbiased treatment allocation.

### Rotarod

Motor coordination of each mouse was assessed using a rotarod device (Rota Rod, BIOSEB). Mice were first placed on the static rotor to acclimate to the apparatus, followed by a trial at a rotation speed of 4 rpm for 60 s. After a 15-min rest, the mice were reacclimated. Training was conducted for 2 d prior to the start of testing. During both training and testing, the rotarod was set to start at 4 rpm, gradually accelerating to 40 rpm over a span of 300 s. The latency to fall was recorded for each mouse over 3 consecutive trials, with a 15-min interval between trials. The results from 3 trials were averaged to assess motor performance. Each mouse was recorded beginning at 8 weeks of age and subsequently every 2 weeks until reaching 16 weeks.

### Spinal cord single-nucleus isolation

Postnatal 115-d-old SOD1^G93A^ mice and their littermates were transcardially perfused with phosphate-buffered saline (PBS), and the entire spinal cord was rapidly dissected. The dissected tissue was snap-frozen in liquid nitrogen and stored at −80 °C. Nuclei were isolated and purified following the 10× Genomics protocols (CG000393•Rev A) with slight modifications. Briefly, the frozen tissues were homogenized on ice in Nuclei EZ lysis buffer (NUC-101; Sigma-Aldrich), supplemented with protease inhibitor (5892791001; Roche) and RNase inhibitor (3335399001; Roche), using a glass homogenizer (885302-0002; Kimble Chase). The tissue was dissociated until no visible clumps remained. The homogenates were incubated on ice for 5 min, and an equal volume of HEB medium (Hibernate E/B27/GlutaMAX, Gibco) was added to halt lysis. Cell debris and large clumps were removed using 30-μm filters (pluriSelect), and the supernatant was centrifuged at 500×g at 4 °C for 5 min. To further remove debris, Debris Removal Solution (130-109-398, Miltenyi) was applied according to the manufacturer’s instructions. Nuclei were washed 1 to 2 times; resuspended in a wash and resuspension buffer containing 1× PBS, 1% bovine serum albumin (BSA, Sigma), and 0.2 U/μl RNase inhibitor; and collected by centrifugation at 500×g for 5 min at 4 °C. The nuclear suspension was then diluted to a concentration of 700 to 1,200 nuclei/μl for loading onto a 10× Chromium instrument.

### Single-nucleus RNA library preparation and sequencing

Nuclear suspension loading onto a Chromium Next GEM Chip, nuclear barcoding, complementary DNA amplification, and library construction were performed following the 10× Genomics protocols (CG000204 Rev D). Libraries were sequenced on the Illumina NovaSeq 6000 System at LCBio Technology Co., Ltd. in Hangzhou, China.

### SnRNA-seq data processing and analysis

The alignment of raw sequencing data to the mouse reference genome (version GRCm38) was conducted using the CellRanger pipeline (version 6.1.2), generating raw gene expression matrices for each sample. The output from CellRanger was loaded into Seurat (version 4.1.1) for dimensional reduction, clustering, and analysis of the snRNA-seq data. DoubletFinder (version 2.0.3; https://github.com/chris-mcginnis-ucsf/DoubletFinder) was used to detect and remove doublet cells with default settings [53].

A total of 44,684 cells passed the QC thresholds: genes expressed in fewer than 3 cells were excluded, cells with fewer than 500 or more than 5,000 genes expressed were also excluded, and cells with unique molecular identifier counts below 500 or with more than 25% of gene_expression derived from mitochondrial_DNA were excluded. Gene expression values were calculated using the NormalizeData() function with normalization.method = “LogNormalize” and scale.factor = 10,000.

To correct for batch effects, we conducted principal component (PC) analysis and employed Harmony to adjust the PCs [54]. Subsequently, we utilized t-distributed stochastic neighbor embedding to project the top 20 Harmony-corrected PCs into 2 dimensions for data visualization and cell type classification. Cell clusters were annotated using markers identified in previous research [19], and 6 main cell types were identified: oligodendrocyte progenitors, neurons, myelinating oligodendrocytes, microglia, endothelial cells, and astrocytes. As for microglia cells, we reclustered them using the above methods and parameters to explore them more thoroughly. Gene signature scores for HOM and DAM were calculated using the UCell R package [55]. The Dimplot(), Dotplot(), and Vlnplot() functions were used to show cell distribution and gene expression levels. Differential expression analysis in different groups was performed by the FindMarkers() function with the Wilcoxon method. Genes expressed in more than 10% of the cells in a cluster, with |log_2_FC| > 0.25 and *P* value < 0.05, were considered statistically significant.

### Gene set enrichment analysis

GO enrichment analysis was performed by GSEA v4.3 (http://www.broadinstitute.org/gsea/) using MSigDB c5 v2023.1 for gene sets ranked by log_2_FC in differential expression analyses. The enrichment score represents the degree of overexpression of a gene set at the top or bottom of the sorted gene list, while the normalized enrichment score was used to estimate GSEA enrichment. Enrichment was considered statistically significant if the *P* value was less than 0.05.

### Pseudotime trajectory analysis

To discover transformational trajectories and characteristic transitions from HOM cells to DAM or cluster 6 cells following SOD1^G93A^, we performed pseudotime analysis and visualization with the Monocle2 package [30]. We first selected HOM cells in the WT and SOD1^G93A^ groups, DAM, and cluster 6 cells as candidate transformational microglia cells. Subsequently, we leveraged the DEGs identified via the differentialGeneTest() function for cell sorting in pseudotime order using Monocle2 [30]. We employed “DDRTree” for dimensionality reduction and applied the BEAM() function along with the plot_genes_branched_heatmap() function for the analysis and visualization of DEGs that depended on each branch.

### Single-cell WGCNA

Single-cell WGCNA was performed by hdWGCNA R package v0.3.1 [56]. The function MetacellsByGroups() was utilized to construct averaged meta cells based on neighboring cells in DAM cells in SOD1^G93A^. Then, we applied SetDatExpr() to aggregate the log-normalized gene expression of nuclei within these DAM cells. To test the optimal soft power threshold parameter between 1 and 30, *n* = 14 was identified using TestSoftPowers() with a signed network. After that, we constructed a coexpression network by ConstructNetwork() with corType = “pearson”. PlotDendrogram(), GetModules(), and GetHubGenes() were used to display the WGCNA dendrogram and module hub genes. Kyoto Encyclopedia of Genes and Genomes pathway enrichment analyses of module genes were performed by the online tool Metascape (http://www.metascape.org).

### Adult microglial cell isolation from the spinal cord and brain stem

Adult microglial cells from C57BL/6 mice were enzymatically dissociated using an Adult Brain Dissociation Kit (130-107-677, Miltenyi Biotec). Spinal cord and brain stem samples (not exceeding 500 mg) were placed in a gentleMACS C tube (130-096-334, Miltenyi Biotec) with 1,950 μl of enzyme mix 1 (enzyme P and buffer Z), to which 30 μl of enzyme mix 2 (enzyme A and buffer Y) was subsequently added. The tube was tightly closed, inverted, and installed onto a gentleMACS Octo Dissociator with Heaters (130-096-427, Miltenyi Biotec), and the appropriate gentleMACS program (37C_ABDK_01) was run. Following the run, the sample was retrieved from the base of the tube, passed through a 70-μm strainer (BS-70-XBS, Biosharp), rinsed in Dulbecco’s PBS, and subjected to centrifugation. The pellet was then resuspended in Dulbecco’s PBS. The myelin and cell debris were segregated by centrifugation of 900×g for 30 min at 18 °C within a 30% to 70% Percoll density gradient. The cell layer at the juncture of the 30% to 70% Percoll layer was collected. Finally, magnetic isolation used anti-CD11b monoclonal-antibody-conjugated microbeads (130-093-636, Miltenyi Biotec) to acquire a pure population of adult microglial cells.

### Real-time PCR

Total RNA was extracted from the whole-spinal-cord samples using a tissue homogenizer and the TRIzol reagent (15596018, Thermo Fisher Scientific) method. RNA was quantified by spectrophotometry using an absorbance of 260 nm, and the sample purity ratios were calculated (260/280 nm). RNA (2 μg) was reverse-transcribed to complementary DNA using PrimeScript RT Master Mix (Takara, RR036A), following the manufacturer’s instructions. Reverse transcription polymerase chain reaction was performed using corresponding primers (Table S13) and TB Green Premix Ex Taq (RR420A, Takara) with Applied Biosystems instruments. All expression values were normalized to housekeeping genes, such as *Gapdh*.

### Immunofluorescence

Immunofluorescence was performed as previously described [16,57]. Cryoprotected tissue was frozen in the optimal cutting temperature compound (SAKURA) and kept at −20 °C until sectioning. Serial sections of the spinal cord, 20 μm thick, were collected on adhering slides (188105, Citotest). Sections were washed with PBS for 10 min and then permeabilized with 0.3% Triton-X 100 in PBS (PBST) for 1 h. Blocking was conducted with 1% BSA (B2064, Sigma-Aldrich) for 1 h at room temperature. The samples were then incubated overnight at 4 °C with primary antibodies diluted in PBST. After 3 washes, the slides were incubated with secondary antibodies for 1 h at 37 °C. Following another 3 washes, the slides were mounted with mounting medium containing 4′,6-diamidino-2-phenylindole (ab104139, Abcam). The primary antibodies used were Iba1 (Wako Catalog [Cat.] No. 019-19741, 1:200; Research Resource Identifier [RRID]: AB_839504), CD11c (Novus Cat. No. NB110-97871, 1:50), GFAP (Abcam Cat. No. ab53554, 1:500; RRID: AB_880202), NeuN (CST Cat. No. 24307, 1:500; RRID: AB_2651140), and choline acetyltransferase (Sigma-Aldrich Cat. No. AB144P, 1:200; RRID: AB_2079751). The secondary antibodies used were goat anti-Armenian hamster-488 (Abcam Cat. No. ab173003), goat anti-rabbit-488 (Thermo Fisher Cat. No. A-11008; RRID: AB_143165), donkey anti-goat-488 (Thermo Fisher Cat. No. A-11055; RRID: AB_2534102), goat anti-rabbit-647 (Thermo Fisher Cat. No. A-21245; RRID: AB_2535813), and donkey anti-goat-594 (Thermo Fisher Cat. No. A-11058; RRID: AB_2534105) and were 1:1,000 applied before capturing images using fluorescence microscopy (Leica, DM6B) and confocal microscopy (Zeiss LSM900).

Image analysis was performed on 3 to 4 microscopic fields in the ventral horn of the lumbar spinal cord for each section. Three sections covering a longitudinal distance of 400 to 600 μm of the spinal cord were analyzed per mouse. The recorded images were loaded into ImageJ (National Institutes of Health) and quantified by 2 independent observers blinded to the group assignments. Positively stained cells were electronically labeled to prevent duplicate counting.

### Flow cytometry

Animals were euthanized and perfused with cold saline, and the whole spinal cords were collected. Spinal cord homogenates were prepared using the Adult Brain Dissociation Kit (Miltenyi Biotec) and a gentleMACS Dissociator with heaters (Miltenyi Biotec) according to the manufacturer’s instructions. The cell suspension was passed through a 70-μm cell strainer (Thermo Fisher Scientific) and resuspended in 30% Percoll. Single-cell suspensions were separated from myelin and debris by centrifugation (900×g, 30 min, acceleration 3, deceleration 1, at 18 °C) on a 30% to 70% Percoll gradient. Cells were collected at the interface of the 30% to 70% Percoll layers and subsequently washed with Hank’s balanced salt solution (Thermo Fisher Scientific) containing 2% fetal bovine serum (Thermo Fisher Scientific). The single-cell samples were then incubated with antibodies against surface antigens CD11b-fuorescein isothiocyanate (BioLegend Cat. No. 101205; RRID: AB_312788; 1:200), CD45-phycoerythrin (BioLegend Cat. No. 103106; RRID: AB_312971; 1:200), and CD11c-biotin (BioLegend Cat. No. 117304; RRID: AB_313773; 1:200) for 30 min at 4 °C in the dark. After a wash, cells were incubated with the secondary antibody APC/Cy7 streptavidin (BioLegend Cat. No. 405208; RRID: AB_466447; 1:200) for 30 min at 4 °C in the dark. Flow cytometry was performed with a Beckman flow cytometer (CytoFLEX LX), and data were analyzed using the FlowJo software.

### RNAscope ISH combined with immunofluorescence

RNAscope ISH was performed on cryosections using RNAscope 2.5 HD Detection Reagents-RED (322360; Advanced Cell Diagnostics) according to the manufacturer’s protocol with some modifications. Briefly, 20-μm sections were mounted onto Superfrost Plus slides (12-550-15; Thermo Fisher Scientific) and were removed from the cryoprotectant by washing 3 times in PBS for 5 min each. Sections were gradually dehydrated in 50%, 70%, and 100% ethanol for 5 min each and air-dried at room temperature. Then, the slides were incubated with protease (322330; Advanced Cell Diagnostics) for 10 min at room temperature and washed twice in DEPC water for 2 min each. Next, lumbar spinal cord sections were incubated for 2 h at 40 °C with ACD probes for mouse *Cx3cr1* (314221; target NM_009987.4, region 58 to 1,073 bp), *P2ry12* (317601; target NM_027571.3, region 739 to 1,854 bp), and *Clec7a* (532061; target NM_020008.3, region 95 to 1,212 bp), followed by washing in 1× RNAscope wash buffer. The amplification and detection reagents containing AMP 1, AMP 2, AMP 3, AMP 4, AMP 5, AMP 6, Fast RED-A, and Fast RED-B were used as recommended. To combine ISH with immunofluorescence staining, the tissue sections were then blocked with PBS with 1% BSA and 0.3% Triton X-100 for 1 h and incubated overnight at 4 °C with anti-Iba1 (Wako Cat. No. 019-19741, 1:100; RRID: AB_839504), GFAP (Abcam Cat. No. ab53554, 1:500; RRID: AB_880202), and NeuN (CST Cat. No. 24307, 1:500; RRID: AB_2651140) primary antibodies. Sections were then incubated with the goat anti-rabbit-488 (Thermo Fisher Cat. No. A-11008; RRID: AB_143165) and donkey anti-goat-488 (Thermo Fisher Cat. No. A-11055, RRID: AB_2534102) secondary antibodies (1:1,000) for 1 h at room temperature and counterstained with 4′,6-diamidino-2-phenylindole before mounting. An image was obtained using confocal laser scanning fluorescent microscopy (Zeiss LSM900) or fluorescence microscopy (Leica, DM6B) using a ×20 objective.

### RNAscope ISH signal quantification

To quantify the colocalization of red and green signals in ISH images, the FIJI software was employed. Images were first converted into grayscale, and individual channels for red and green signals were separated. The “Threshold” tool was applied to each channel to define the signal regions, followed by the “Analyze Particles” function to count individual red and green signals. For merged signals, a combined image was generated using the “Merge Channels” function, and the number of colocalized spots (merge) was manually counted using the “Cell Counter” plug-in. The percentage of merged signals was calculated by dividing the number of colocalized signals by the total number of red or green signals and multiplying by 100%.

### Pearson’s correlation coefficient calculation

To verify colocalization, images were first converted to 16-bit format. The Colocalization Finder plug-in was then selected, and the 2 channels of interest were loaded for colocalization analysis. Appropriate settings for colocalization parameters were confirmed, and the output provided the Pearson’s correlation coefficient, enabling a quantitative assessment of the colocalization level.

### Statistical analyses

All data are reported as mean values ± standard error of the mean unless otherwise indicated. A Student *t* test was used to compare outcomes between 2 groups. Statistical analyses were conducted using GraphPad Prism v8.4.3. All statistical tests were 2-sided. The statistical tests used for each analysis, the sample size, and the significance levels are reported in the caption of each figure.

## Data Availability

The raw single-cell files for each animal are available in the National Genomics Data Center (NGDC) database, with accession number CRA018998. All software and algorithms used in this study are publicly available and listed in Table S14.
