# Supplementary material for: Single-Nucleus RNA Sequencing Reveals the Spatiotemporal Dynamics of Disease-Associated Microglia in Amyotrophic Lateral Sclerosis
Source: Research (Wash D C). 2024 Dec 11;7:0548. doi: 10.34133/research.0548 (PMC11632836; doi:10.34133/research.0548)
Supplement: Supplementary 1 — Figs. S1 to S9 Tables S1 to S14 [file research.0548.f1.zip › Supplementary information.docx]

***Legends***

***Fig. 1.*** SnRNA-seq of spinal cords in SOD1^G93A^ mouse model of ALS. (A) Schematic summarizing design: whole spinal cords from postnatal day 115 SOD1^G93A^ and WT littermate male mice (*n* = 2 / group) were prepared and sequenced. (B) t-Distributed stochastic neighbor embedding (tSNE) plot of cell types in two groups. (C) Dot plot of canonical marker genes^19^ for identified cell types. (D) Bar plots showing the proportion of each cell type. (E) Bar plot displaying the Top 20 Gene Ontology (GO) pathways analyzed by Gene Set Enrichment Analysis (GSEA) enriched in differentially expressed genes (DEGs) between SOD1^G93A^ and WT group. (F) tSNE plots of cells in WT and SOD1 ^G93A^ mice. (G) Proportion of each cell type separated by two groups. The number of microglia in the SOD1^G93A^ group is much higher than that in the WT group.

***Fig. 2.*** Disease-associated microglia show a dramatic increase in SOD1^G93A^ mice. (A) tSNE plot highlights the microglia in color blue. (B) tSNE plot showing 9 subclusters of microglia population with “resolution = 0.8”. (C) Stacked bar graph showing the average percentages of cells in each cluster of microglia in WT and SOD1^G93A^ mice. (D) Comparison of the proportions of microglia subpopulations in in WT and SOD1^G93A^ mice. The number of cells in C5 and C6 of the SOD1^G93A^ group is higher than that in the WT group, especially in cluster 5. (E) Bar plots showing the expression level of six homeostatic microglia (HOM) markers in C0 compared to all other microglia subclusters. ***P* value < 0.01, *****P* value < 0.0001, C0 vs. others. (F) t-SNE plot showing subcluster annotations of microglia. The numbers of microglia in WT and SOD1^G93A^ are in parentheses. (G) Volcano plot showing the DEGs between disease-associated microglia (DAM) to HOM. Blue dots represent down-regulated DEGs, and red dots represent up-regulated DEGs. Genes related to DAM and HOM signature were labeled by yellow. (H) GO enrichment analysis was performed by GSEA on genes ranked by log2FC between DAM and HOM. Bar plot showing 4 major pathway categories: “steroid regulation/ foam cell differentiation” colored by pink, “skeletal muscle regeneration” colored by orange, “cell adhesion/ endocytosis” colored by green, and “immune/ inflammatory response” colored by blue. (I) The relationship between the activated GO terms in Fig.2H and the core genes are depicted. Circos plots showed the core genes related to “cell adhesion/ endocytosis”, “immune/ inflammatory response”, “skeletal muscle regeneration” and “steroid regulation/ foam cell differentiation” function.

***Fig. 3****.* Cluster 6 microglia show a dramatic increase in SOD1^G93A^ mice. (A) Volcano plot showing the DEGs between cluster 6 microglia and HOM. Blue dots represent down-regulated DEGs, and red dots represent up-regulated DEGs. (B) GO enrichment analysis was performed on genes ranked by log2FC between cluster 6 microglia and HOM. Bar plot showing 3 major pathway categories: “synapse assembly”, “axonogenesis”, and “neurogenesis”. (C) The relationship between the activated GO terms in (B) and the involved genes is depicted. Circos plot showed the core genes related to the pathways. (D) Bar plot displaying the Top 10 GO enrichment pathways in cluster 6 between SOD1^G93A^ mice and WT mice.

***Fig. 4.*** Disease-associated microglia evolve from homeostatic microglia and are conserved from mouse to human neuropathology in ALS. (A) Pseudotime analysis of single-cell trajectory of HOM, DAM and cluster 6 microglia using Monocle2. Two major branches were identified, one led to cluster 6 cells in SOD1 ^G93A^ from HOM (Cell fate1), the other led to DAM in SOD1^G93A^ (Cell fate 2). (B) Representative genes related to HOM and DAM signatures were selected to show their expression trends before and after cell state branching. (C) Gene signatures during microglia state switch. The expression level of DEGs (rows) are shown using heatmap with microglia (columns) in pseudotime from Root to Cell fate 1 or Cell fate 2. Gene-expression trends in each group (middle). GO terms associated with DEGs in the four kinetic clusters (left/right).

(D-E) Weighted gene coexpression network analysis (WGCNA) was performed in DAM and identified two gene modules were enriched. Network plots of the top 25 genes with the highest intramodular connectivity in yellow and camel modules. (F) KEGG pathways enriched by DAM-M12. Circos plot showed genes related to these KEGG pathways of “Huntington disease”, “Amyotrophic lateral sclerosis”, and “Neurodegeneration – multiple diseases”. (G) Heatmap showing the markers of DAM and HOM of frontal cortex of healthy controls, sporadic ALS and *C9orf72* mutated ALS (GSE67196).

***Fig. 5.*** DAM appear in the spinal cord and brainstem after motor neuron death in SOD1^G93A^ mice. (A-B) Representative microscopic images and quantification of NeuN^+^ and ChAT^+^ motor neurons at ventral horn of lumbar spinal cord in SOD1^G93A^ and WT mice at different time points stained for NeuN. Nuclei are labeled with DAPI. Scale bar: 20 μm. (C) Weekly rotarod performances in WT (n = 14), SOD1 (n = 6) mice. Mean holding times on the rotating rod at indicated weeks were plotted. **p* < 0.05 and ***p* < 0.01, Student’s *t*-tests. Data are mean ± sem. (D-I) Quantitative RT-qPCR analysis of DAM signatures in cerebrum, brainstem, spinal cord at P60 and P110. Each result was normalized to *Gapdh*. Error bars denote sem (n = 3-6). **p* < 0.05, ***p* < 0.01, ****p* < 0.001, *****p* < 0.0001, Student’s *t*-tests. (J) A line chart showing average fold change of temporal evolution of each DAM marker.

***Fig. 6.*** DAM exhibit an increase during disease progression in SOD1^G93A^. (A) Flow cytometry analysis of CD11c^+^CD11b^+^CD45^+^ DAM in the spinal cord (per 10^4^ total microglia) at 90 days, 110 days, 130 days, 150 days, and 170 days in SOD1^G93A^ and WT mice. (B) Statistics of percent of CD11c^+^ DAM at different time points. **p* < 0.05 and ***p* < 0.01, Student’s *t*-tests. (C-E) Fresh-frozen spinal cords from 110 days WT and SOD1^G93A^ littermates were sectioned and prepared using RNA-scope with the probes (*Cx3cr1*, *P2ry12* and *Clec7a*). Scale bar, 75 μm. (F-H) Quantification of (C-E) percent of mRNA probes positive microglia analyzed per image. n = 3 animals per group; n = 3 to 4 images from ventral horn (1 image per section). **p* < 0.05; ****p* < 0.001; ns, not significant; Student’s *t*-tests. Data are represented as mean ± sem. (I) Representative images from lumbar spinal cord in SOD1^G93A^ mice and their littermates at 110 days and 150 days, stained for a DAM marker gene (CD11c, green), Iba1-1 (microglia, red), and DAPI (cell nuclei, blue). Scale bar, 100 μm. (J) Quantification of percent of CD11c^+^ microglia analyzed per image. n = 3 animals per group; n = 3 to 4 images from ventral horn (1 image per section). ****p* < 0.001, *****p* < 0.0001, Student’s *t*-tests. (K) Colocalization analysis of mRNA (*Cx3cr1*, *P2ry12* and *Clec7a*) and protein (CD11c) with microglia (Iba1) using Pearson's correlation coefficient (Pearson’s R). Pearson’s R above 0.5 typically indicates a significant level of colocalization.

***Fig. 7.*** DAM are independent of CSF1R for survival. (A) Schematic summarizing design: 2-month WT mice were fed with or without PLX5622 for 4 days and subsequently sacrificed for immunofluorescence and flowcytometry. (B) Representative microscopic images of lumbar spinal cord staining Iba1 for microglia, GFAP for astrocytes, NeuN for neurons in WT mice with or without PLX5622. Scale bar, 100 μm. (C-E) Quantitative percentage of microglia, astrocytes and neurons between vehicle and PLX5622 in WT mice. n = 3 animals per group; n = 3 to 4 images from ventral horn (1 image per section). (F) Flow cytometry analysis showing the percentage of microglia (CD11b^+^CD45^+^). The top right panel shows microglia from control mice, while each scatter plot in the bottom row corresponds to an individual mouse treated with PLX5622 for 4 days. (G) Schematic summarizing design: 3-month SOD1^G93A^ mice were fed with or without PLX5622 for 7 days and subsequently sacrificed for flowcytometry. (H) Flow cytometry analysis of CD11b^+^CD45^+^ microglia and CD11c^+^CD11b^+^CD45^+^ DAM between vehicle and PLX5622 in SOD1^G93A^ mice. (I) Quantitative percent of DAM of microglia after using of PLX5622. ***p* < 0.01, Student’s *t*-tests.

***Fig. 8.*** The phagocytic capacity of DAM is enhanced. (A) Schematic summarizing design: brainstem and spinal cord were isolated from SOD1^G93A^ mice and digested into single cells using a gentle MACS dissociator. Adult microglia were sorted by CD11b^+^ magnetic beads and cultured in the plates for 4 days, subsequently performed phagocytosis assay. (B) Confocal images of pHrodo red beads in CD11c^+^Iba1^+^ (DAM) and CD11c^-^Iba1^+^ (the other microglia) at 30 minutes and 60 minutes. Scale bars: 20 μm. (C) Quantitative of pHrodo red beads per microglia (CD11c^+^Iba1^+^ vs CD11c^-^Iba1^+^) at 30min and 60min respectively. Average 20 microglia were counted per well (n = 3). ****p* < 0.001, Student’s *t*-tests.

***Fig. S1****.* (A) Violin plot indicating three QC indicators before filtration: number of detected genes (nFeature_RNA), mRNA counts (nCount_RNA), and mitochondria gene percentage (percent.mt). (B) Violin plot indicating QC indicators after filtration.

***Fig. S2****.* (A) FeaturePlot of canonical marker genes for identified cell types. (B) Violin plot of canonical marker genes for identified cell types. (C) Bar plots showing the proportion of each cell type in individual animals. (D) t-Distributed stochastic neighbor embedding (tSNE) plot of cell types in individual animals.

***Fig. S3****.* (A) Evaluating DAM (including stage 1 and 2) and HOM signatures using UCell. (B) Violin plot showing gene expression level of DAM markers (*Trem2*, *Cd9*, *Ctsd*, *Apoe*, *Csf1* and *Ctsb*) among subclusters of microglia.

***Fig. S4****.* (A) Quantitative RT-qPCR analysis of *Tmod2* and *Trappc9* in spinal cord at P110 and P130. Each result was normalized to *Gapdh*.

***Fig. S5****.* (A) Dotplot showing the relative expression of genes responsible for immune response to interferon (x axis) across all subclusters of microglia (y axis). (B) Dotplot showing the relative expression of genes responsible for immune response to lipopolysaccharide (x axis) across all subclusters of microglia (y axis). (C) Dotplot showing the relative expression of genes responsible for cell cycle (x axis) across all subclusters of microglia (y axis).

***Fig. S6.*** (A) Dot plot displaying average gene expression level in each gene modules of DAM. (B) WGCNA screening for modules relating to DAM. (C) Determination of the soft-thresholding power in the WGCNA analysis.

***Fig. S7****.* (A) Quantitative RT-qPCR analysis of DAM signatures in spinal cord at P90. Each result was normalized to *Gapdh*. (B) Quantitative RT-qPCR analysis of DAM signatures in spinal cord at P130. (C) Quantitative RT-qPCR analysis of DAM signatures in spinal cord at P150. (D) Quantitative RT-qPCR analysis of DAM signatures in spinal cord at P170. Each result was normalized to *Gapdh*. Error bars denote sem (n = 3-6). **p* < 0.05, ***p* < 0.01, ****p* < 0.001, *****p* < 0.0001, ns, not significant, Student’s *t*-tests.

***Fig. S8****.* (A) Quantitative RT-qPCR analysis of DAM signatures in spinal cord at P110. Each result was normalized to *Gapdh*. n = 3-6 animals per group (including male and female animals). (B) Flow cytometry analysis of CD11c^+^CD11b^+^CD45^+^ DAM in the spinal cord (per 10^4^ total microglia) at 130 days in male and female SOD1^G93A^ and WT mice. (C) Statistics of percent of CD11c^+^ DAM at 130 days in male and female SOD1^G93A^ and WT mice. ns, not significant, *****p* < 0.0001, Student’s *t*-tests.

***Fig. S9****.* (A-C) RNA-scope ISH with probes (*Cx3cr1*, *P2ry12* and *Clec7a*), coupling with Immunofluorescence labelling for neurons (NeuN) and astrocytes (GFAP) in the ventral spinal cord at P110 in SOD1^G93A^ and WT mice. Scale bar, 75 μm. (D) Colocalization analysis of mRNA (*Cx3cr1*, *P2ry12* and *Clec7a*) with neurons (NeuN) and astrocytes (GFAP) using Pearson's correlation coefficient (Pearson’s R). Pearson’s R above 0.5 typically indicates a significant level of colocalization.
