# Supplementary figures and images for: Single-Nucleus RNA Sequencing Reveals the Spatiotemporal Dynamics of Disease-Associated Microglia in Amyotrophic Lateral Sclerosis
Source: Research (Wash D C). 2024 Dec 11;7:0548. doi: 10.34133/research.0548 (PMC11632836; doi:10.34133/research.0548)

A

## Before QC filtration

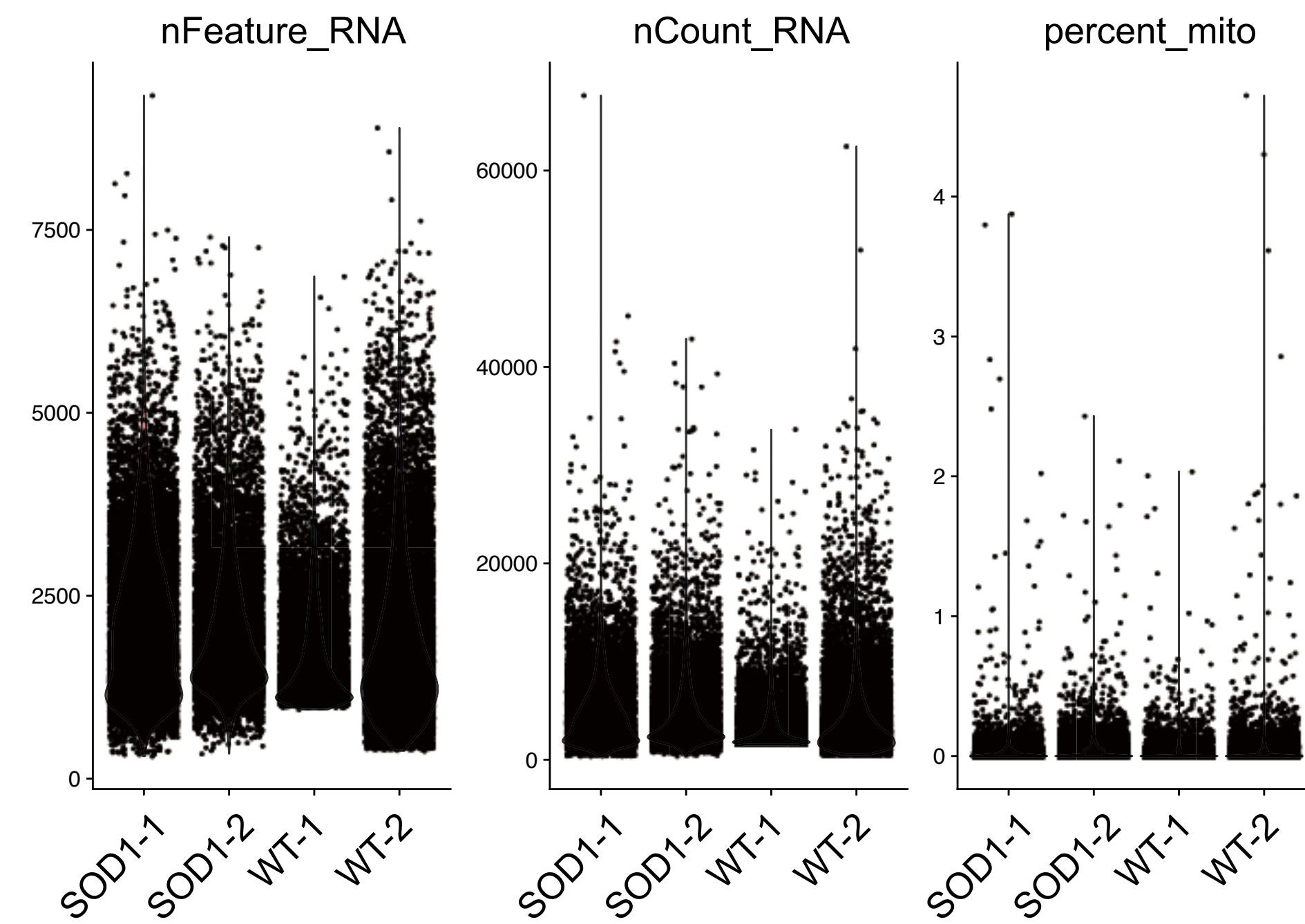

B

## After QC filtration

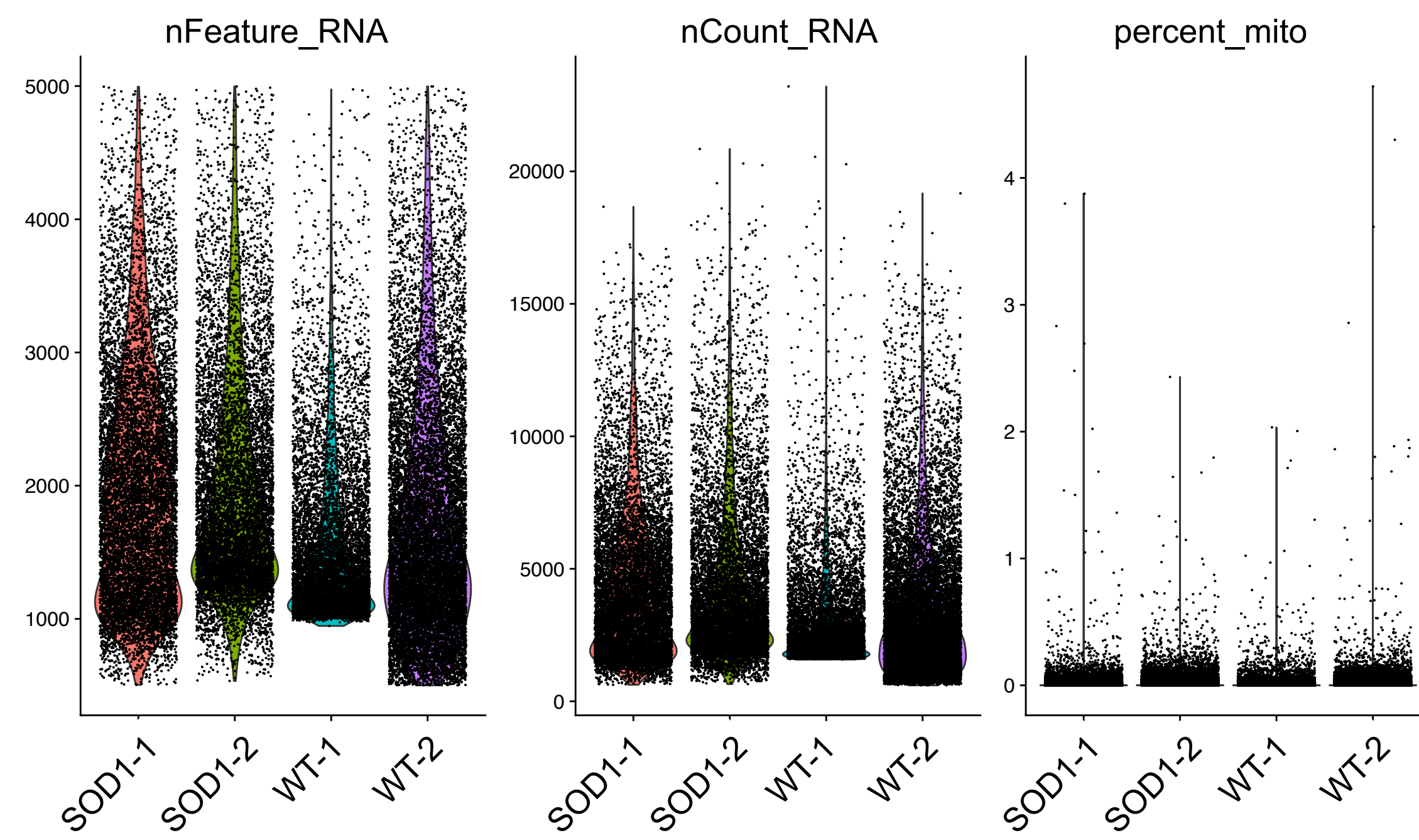

Supplement: Supplementary 1 — Figs. S1 to S9 Tables S1 to S14 [file research.0548.f1.zip › Figure S1.pdf]

A

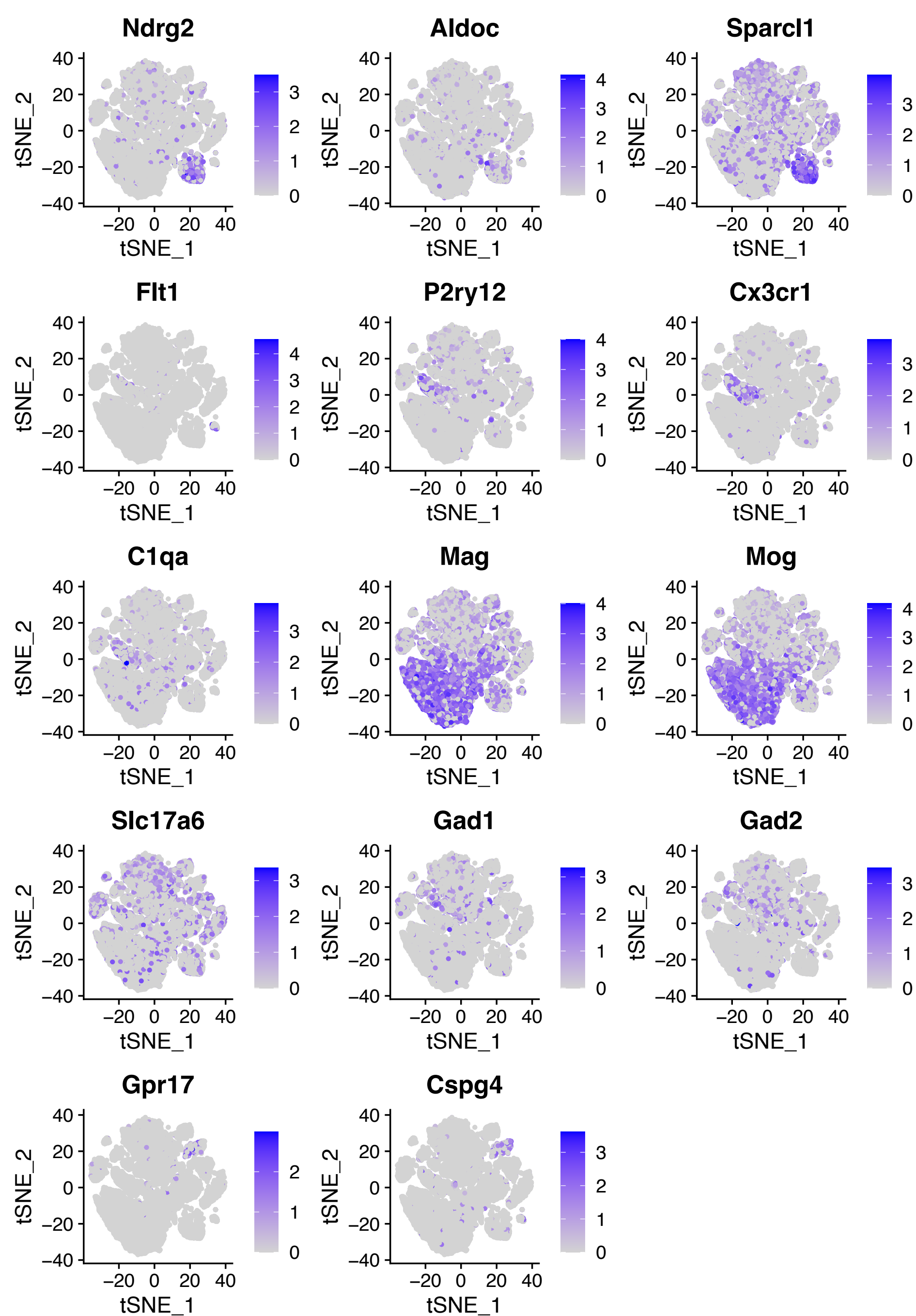

B

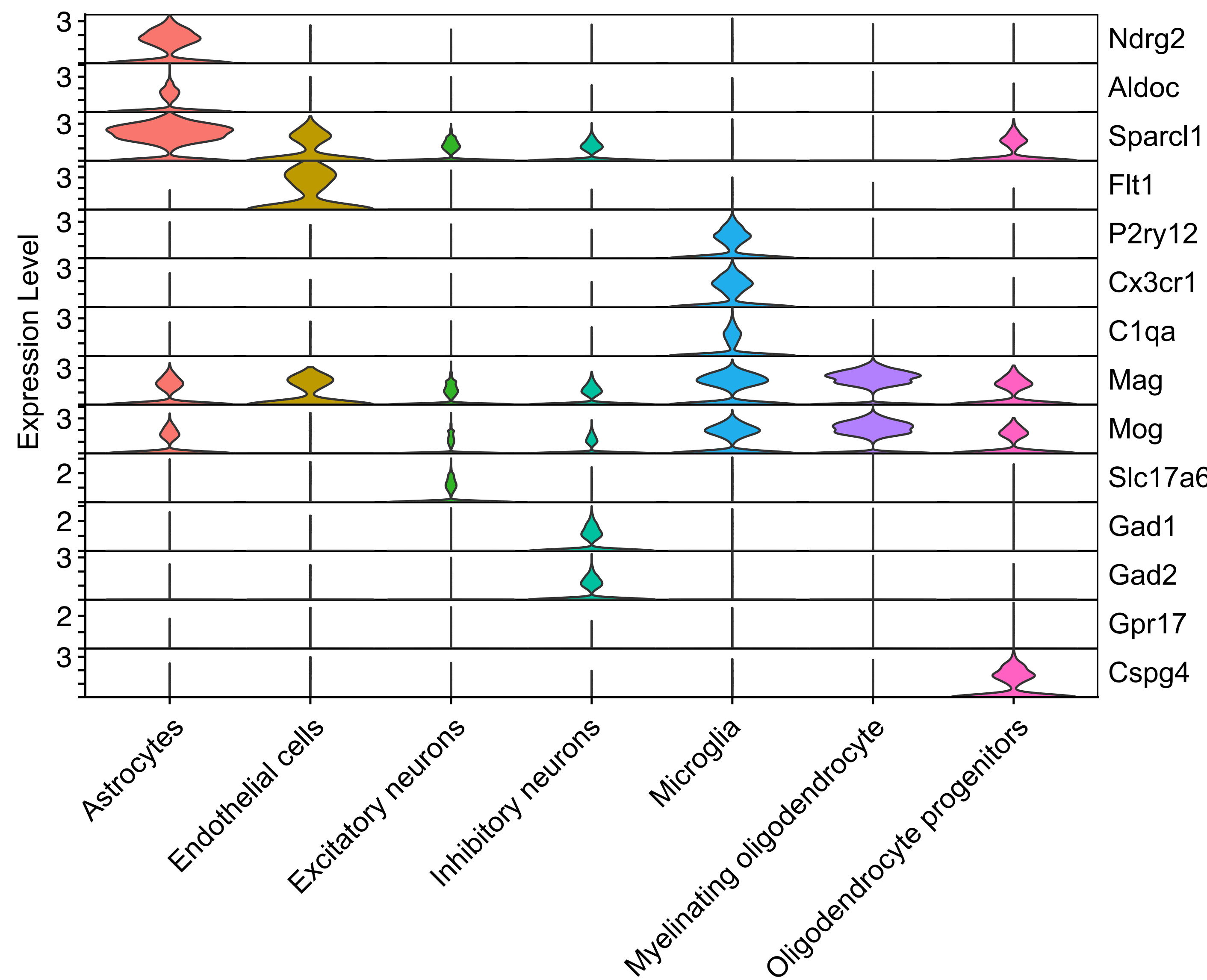

C

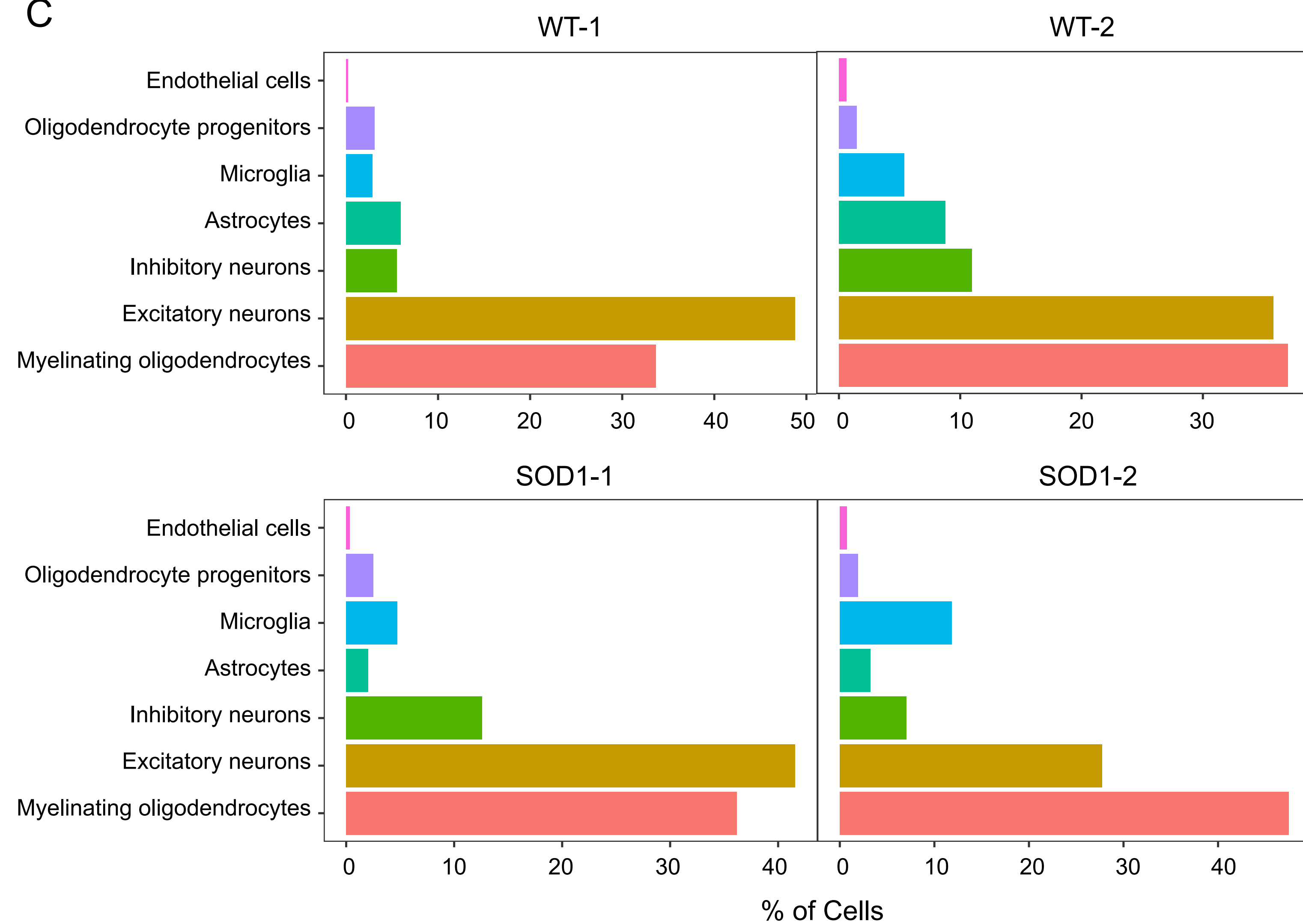

D

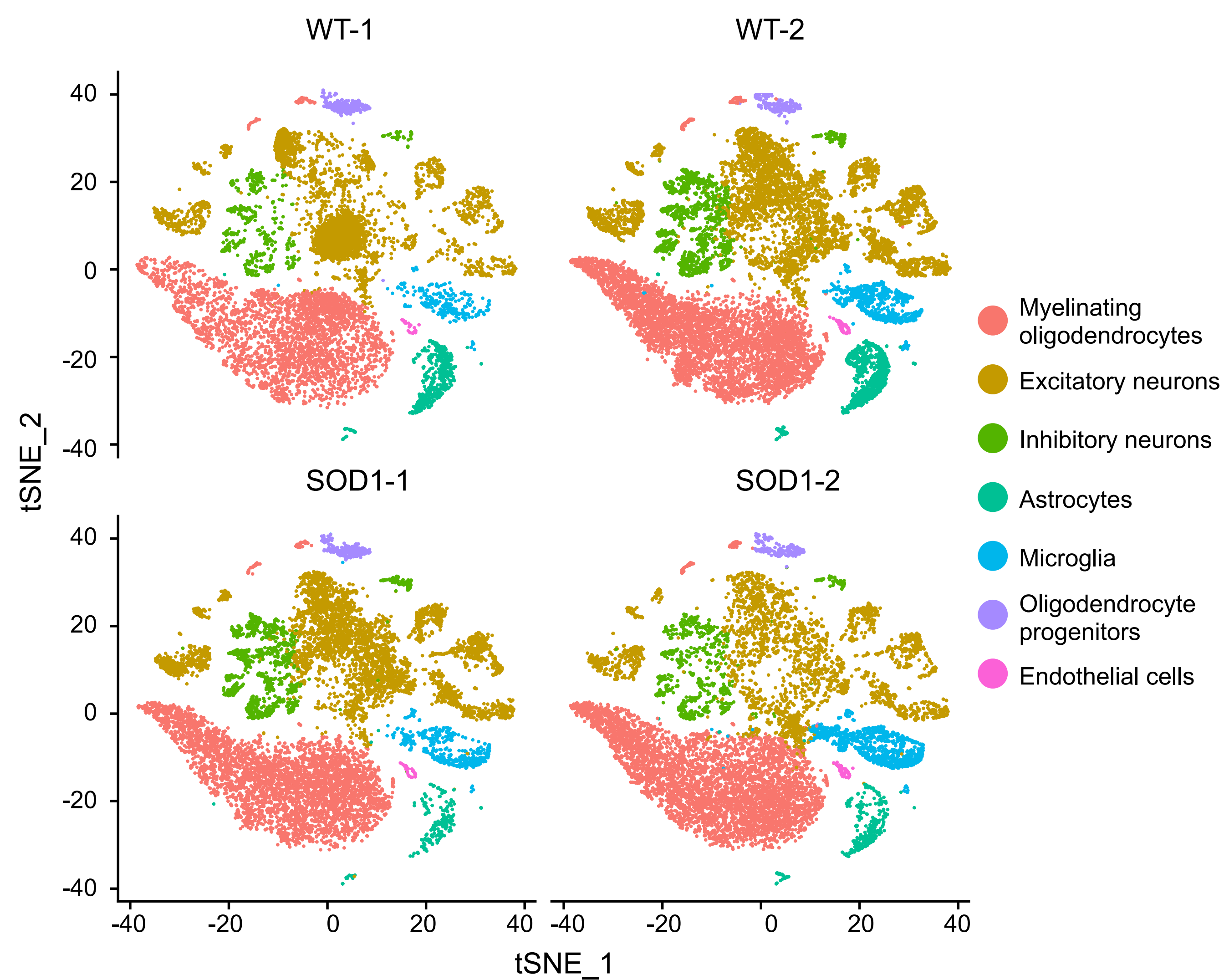

Supplement: Supplementary 1 — Figs. S1 to S9 Tables S1 to S14 [file research.0548.f1.zip › Figure S2.pdf]

A

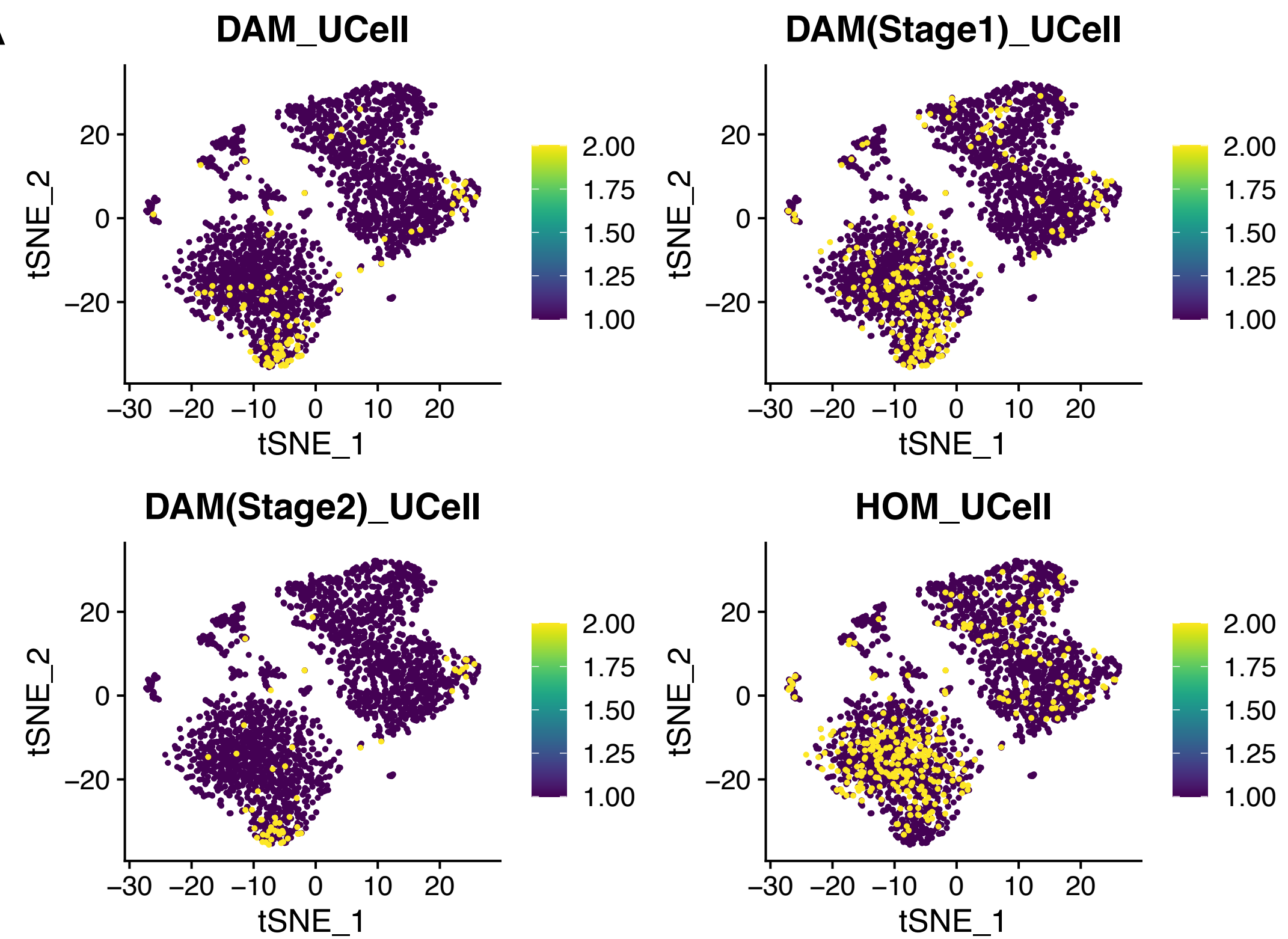

B

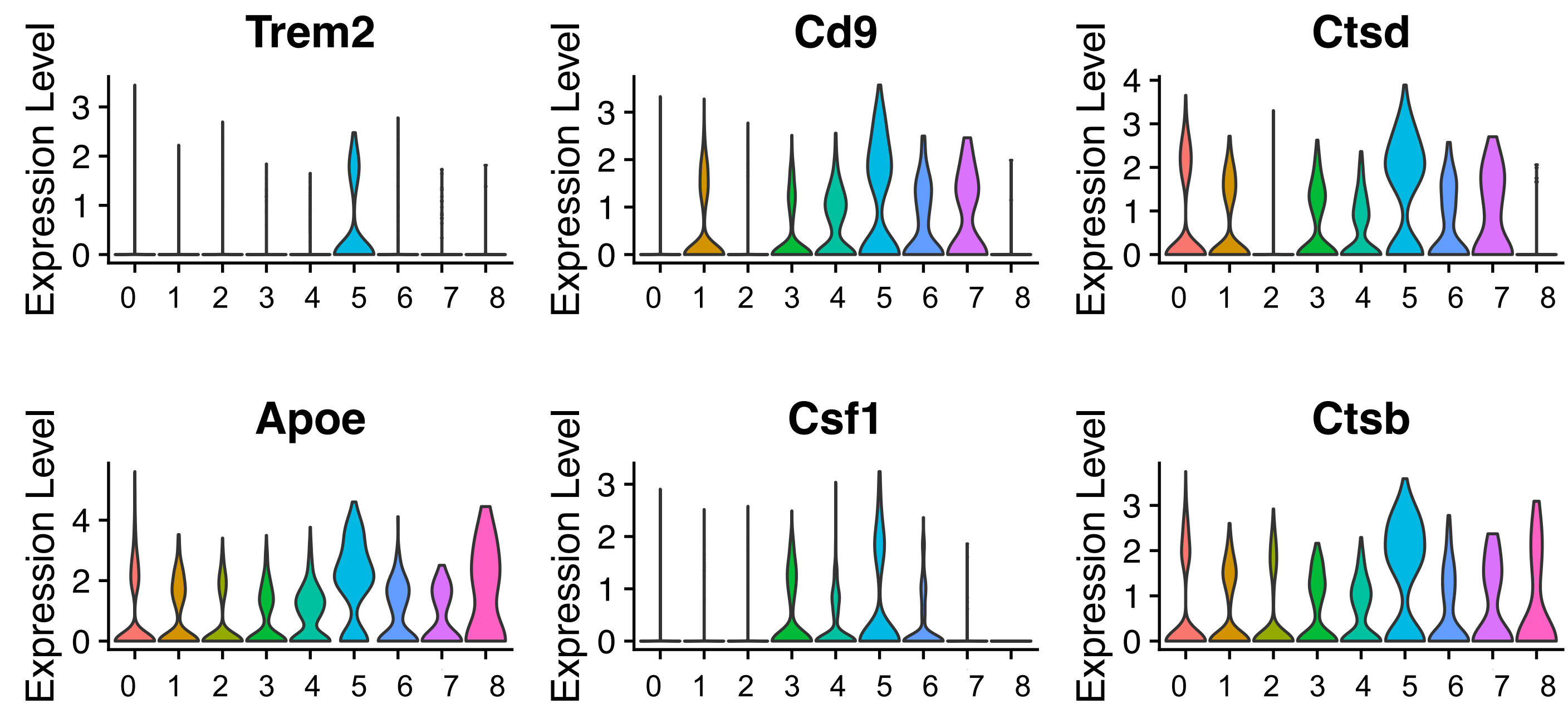

Supplement: Supplementary 1 — Figs. S1 to S9 Tables S1 to S14 [file research.0548.f1.zip › Figure S3.pdf]

A

P110 Spinal cord

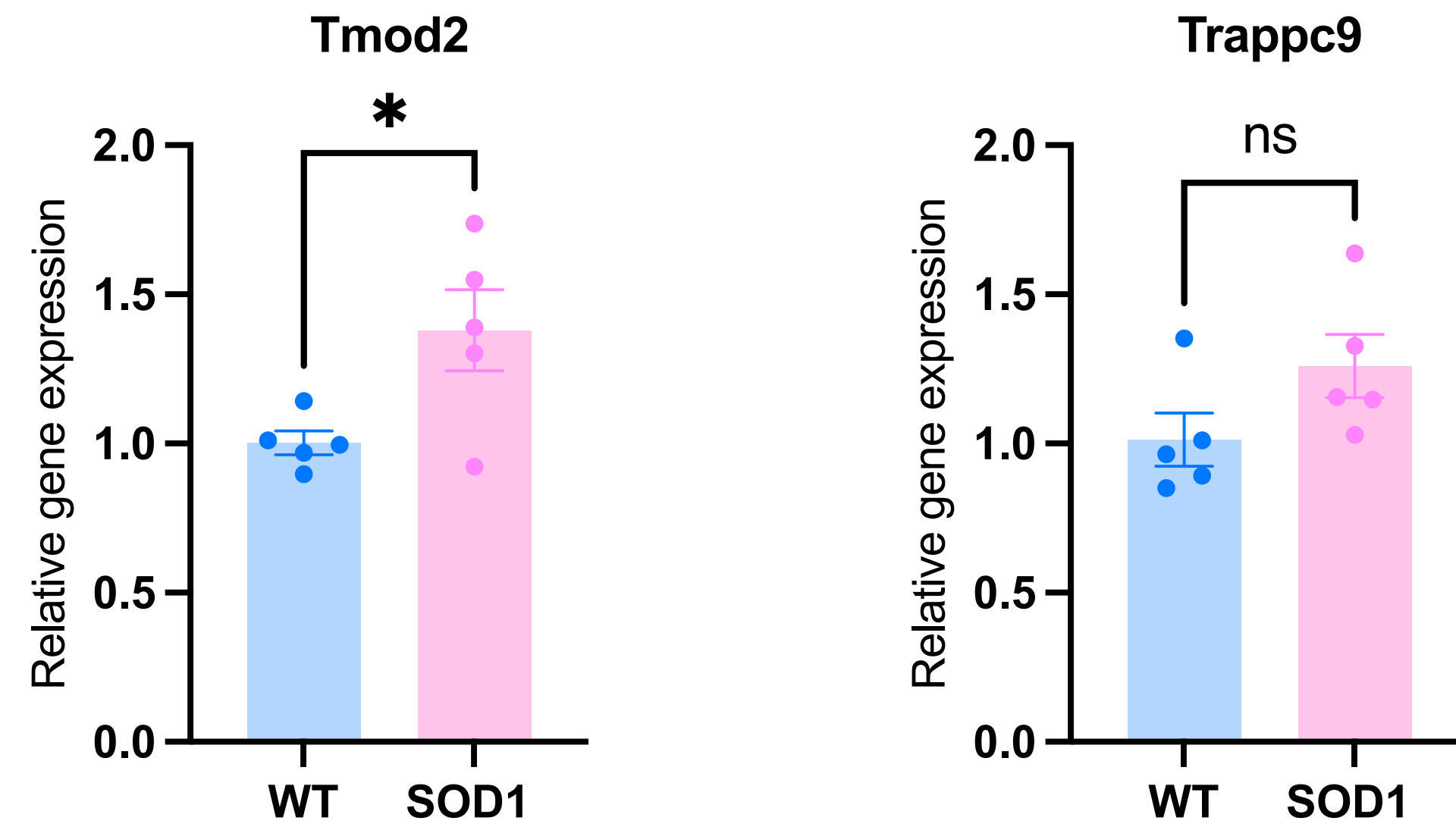

P130 Spinal cord

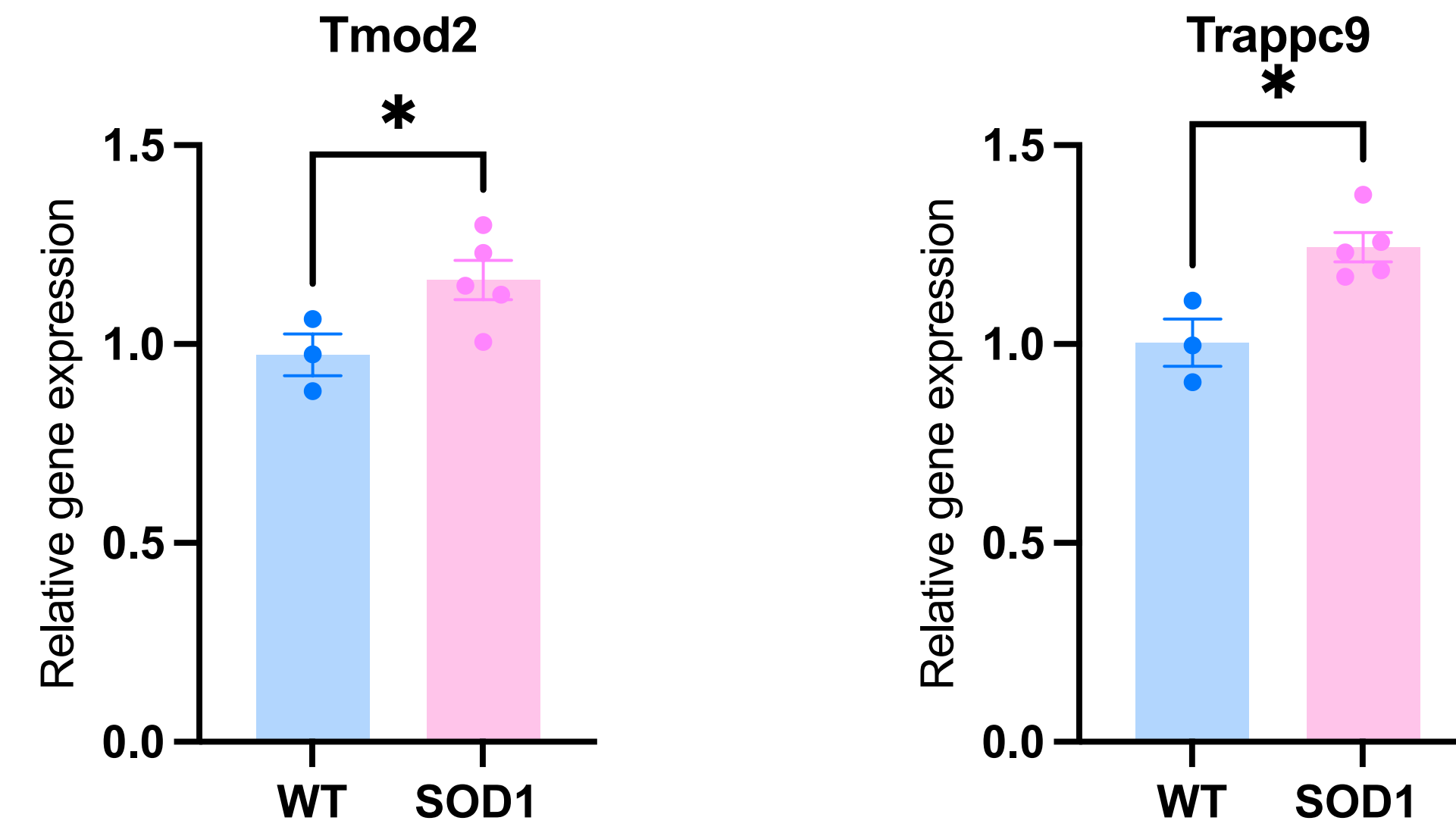

Supplement: Supplementary 1 — Figs. S1 to S9 Tables S1 to S14 [file research.0548.f1.zip › Figure S4.pdf]

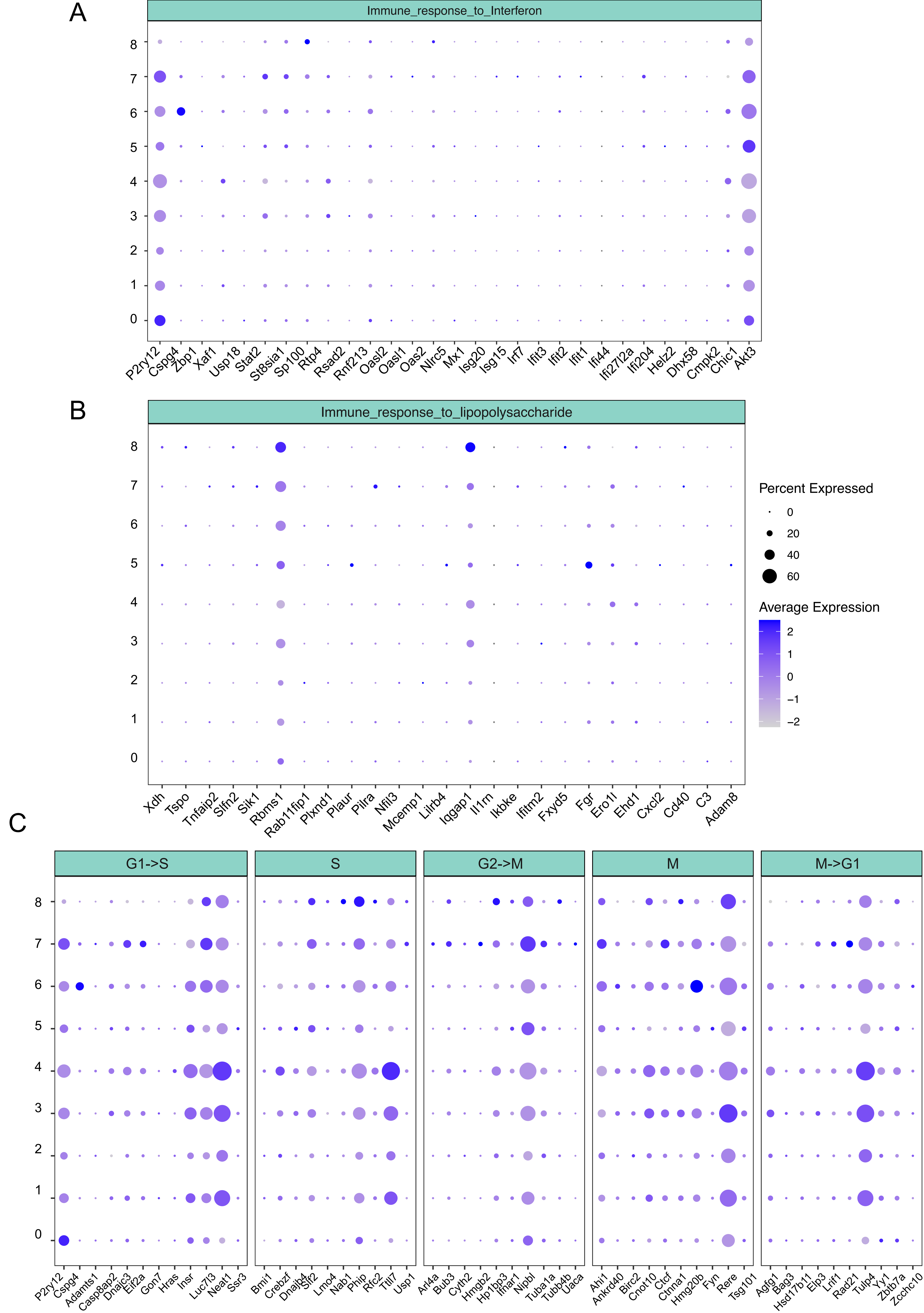

Supplement: Supplementary 1 — Figs. S1 to S9 Tables S1 to S14 [file research.0548.f1.zip › Figure S5.pdf]

A

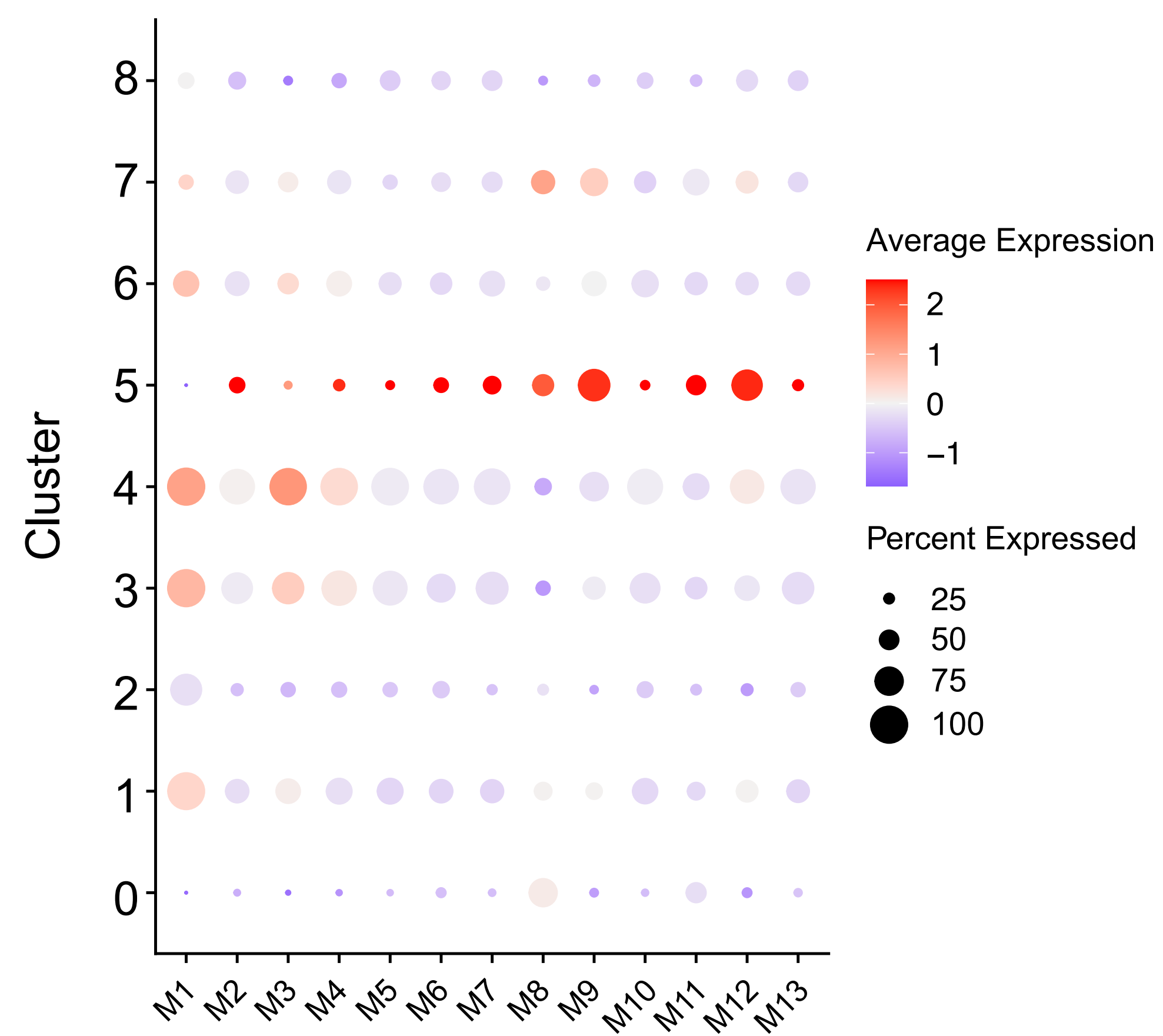

B

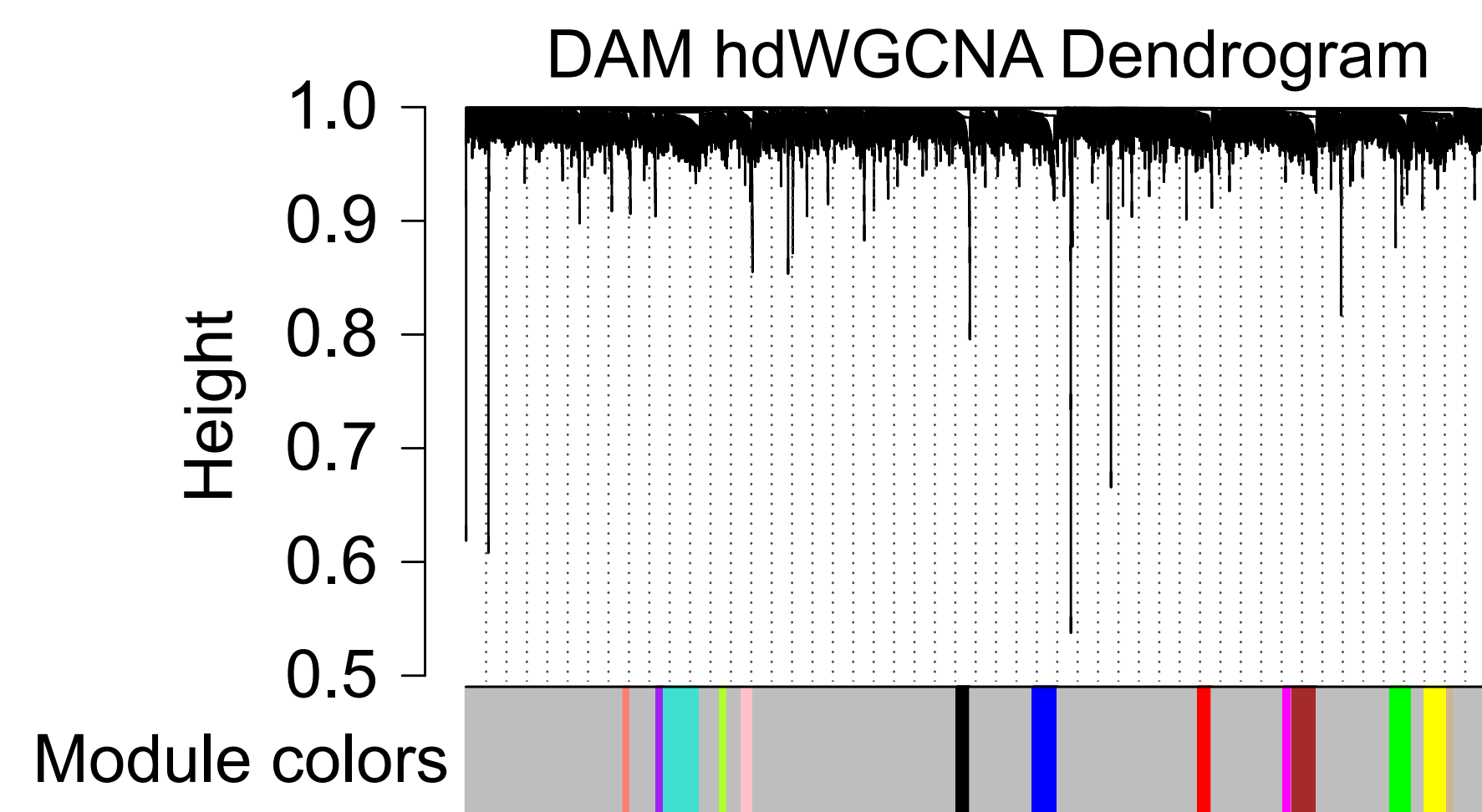

C

Scale-free Topology Model Fit

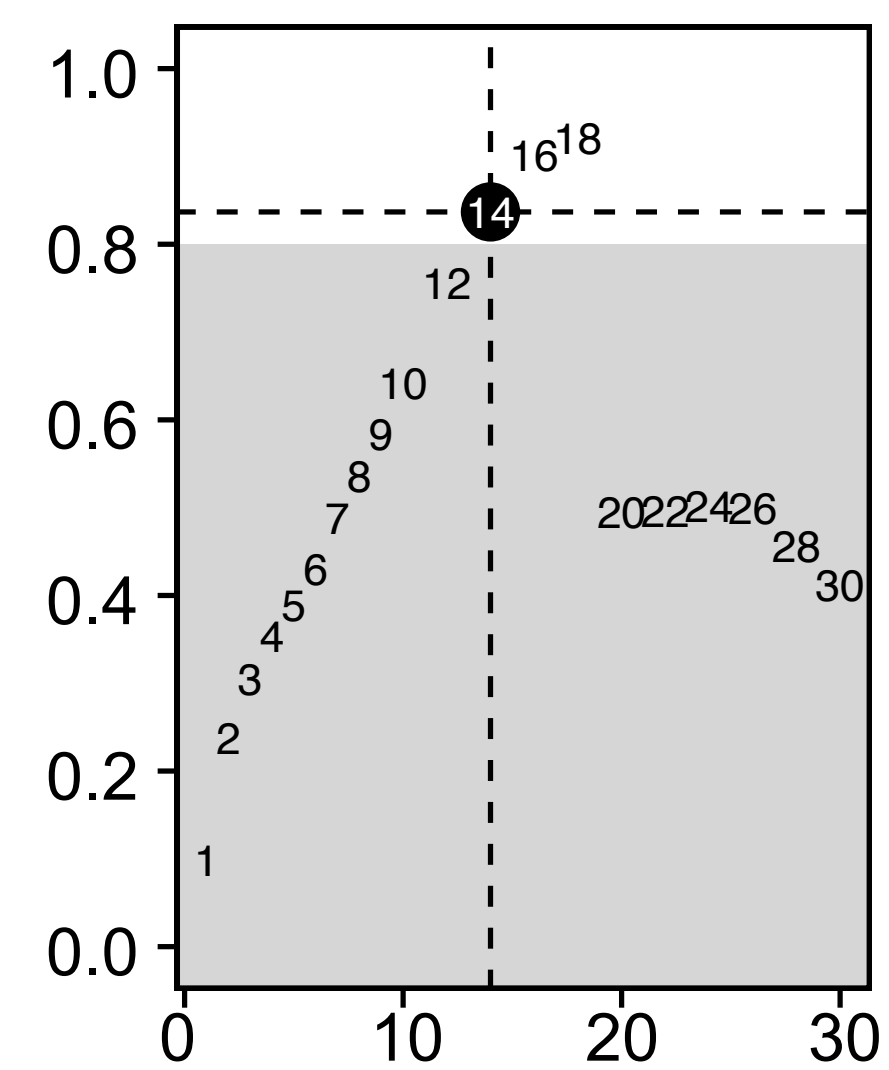

Mean Connectivity

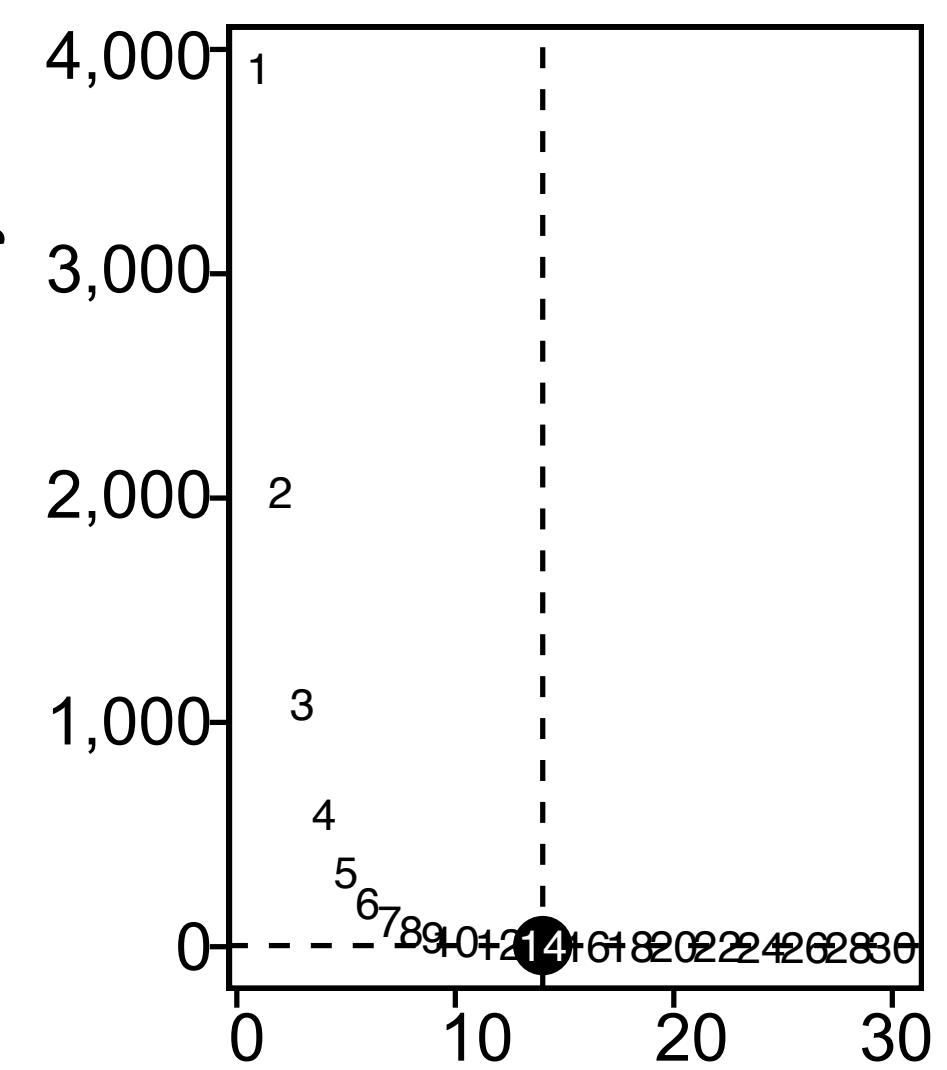

Median Connectivity

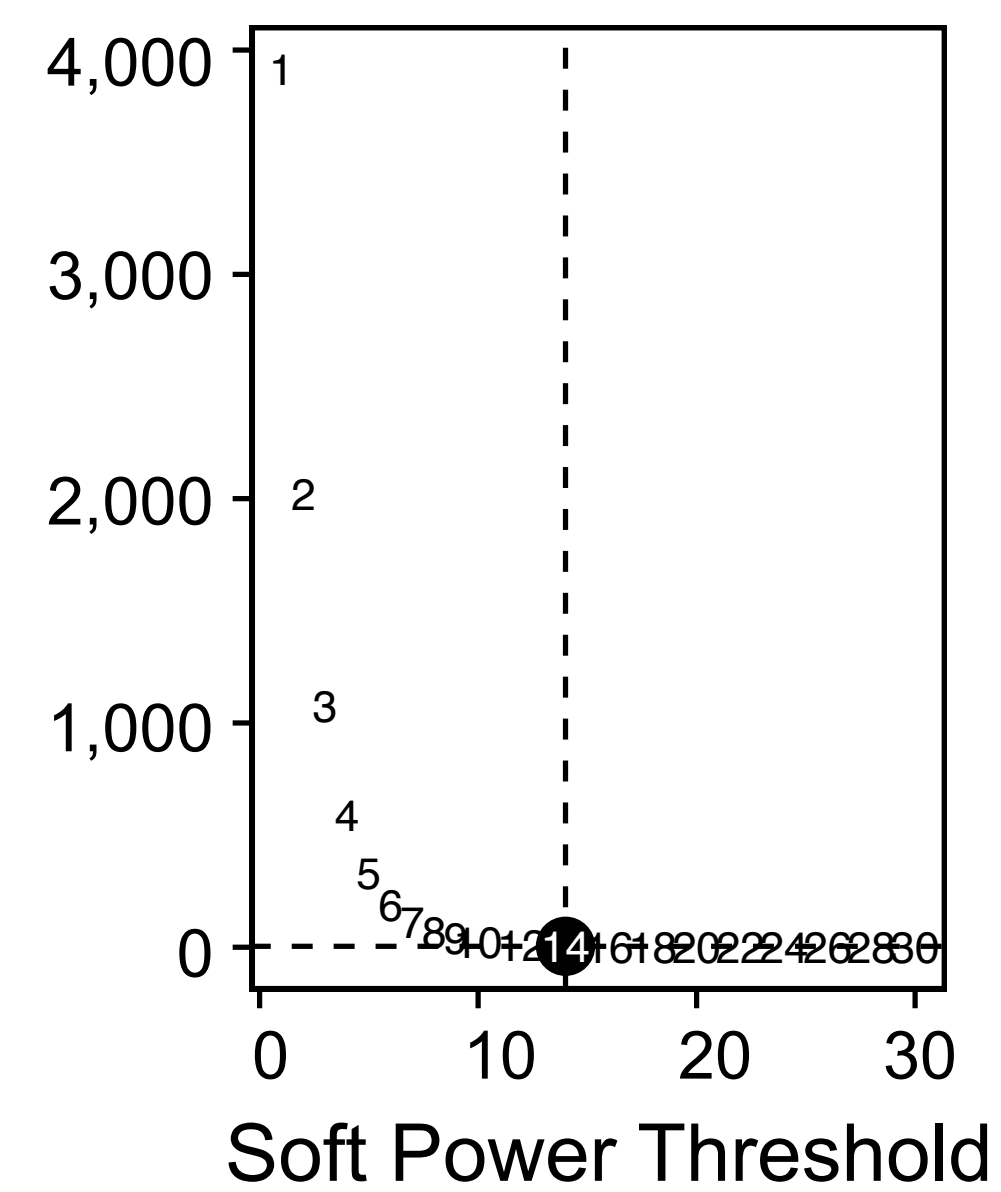

Max Connectivity

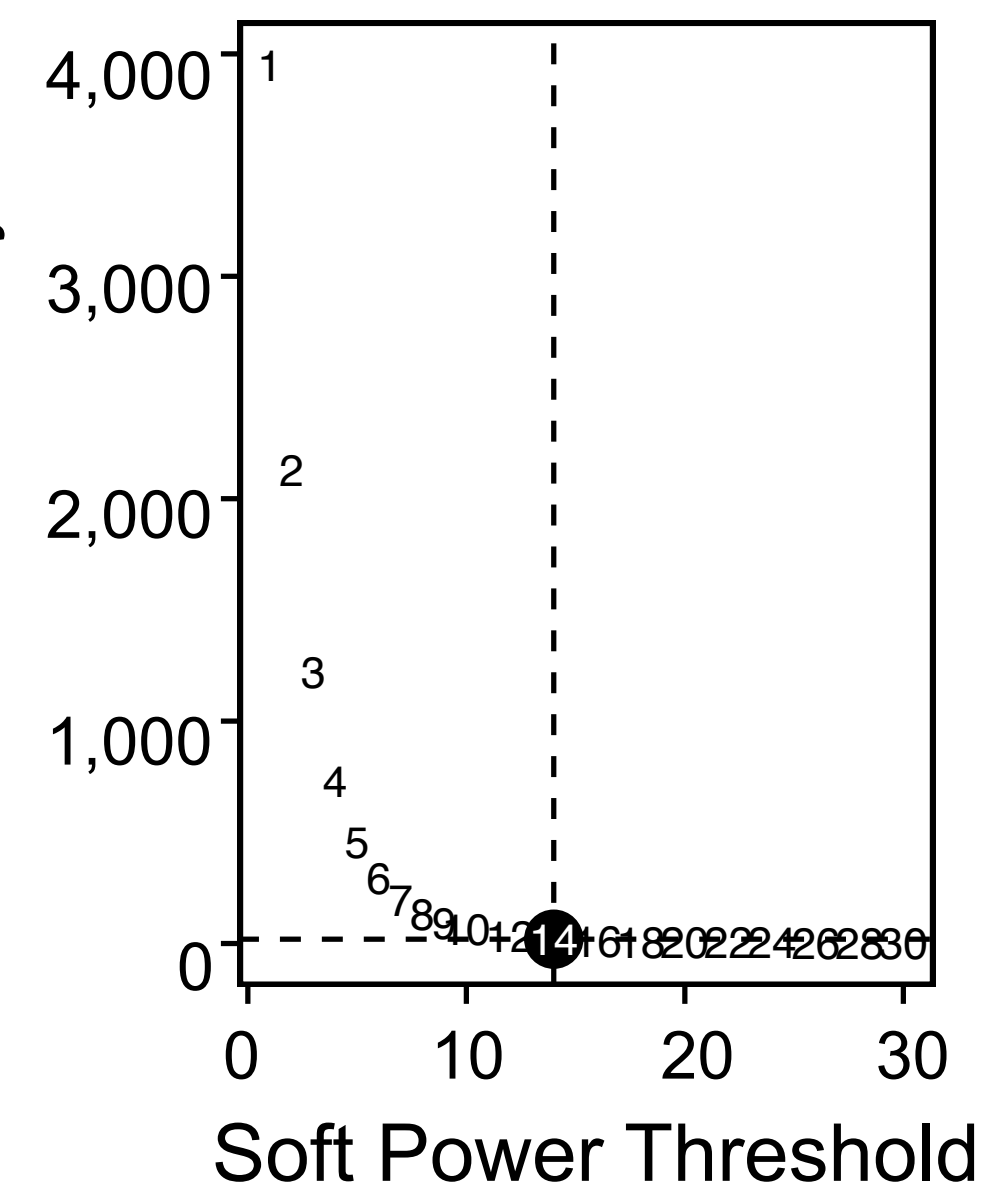

Supplement: Supplementary 1 — Figs. S1 to S9 Tables S1 to S14 [file research.0548.f1.zip › Figure S6.pdf]

A

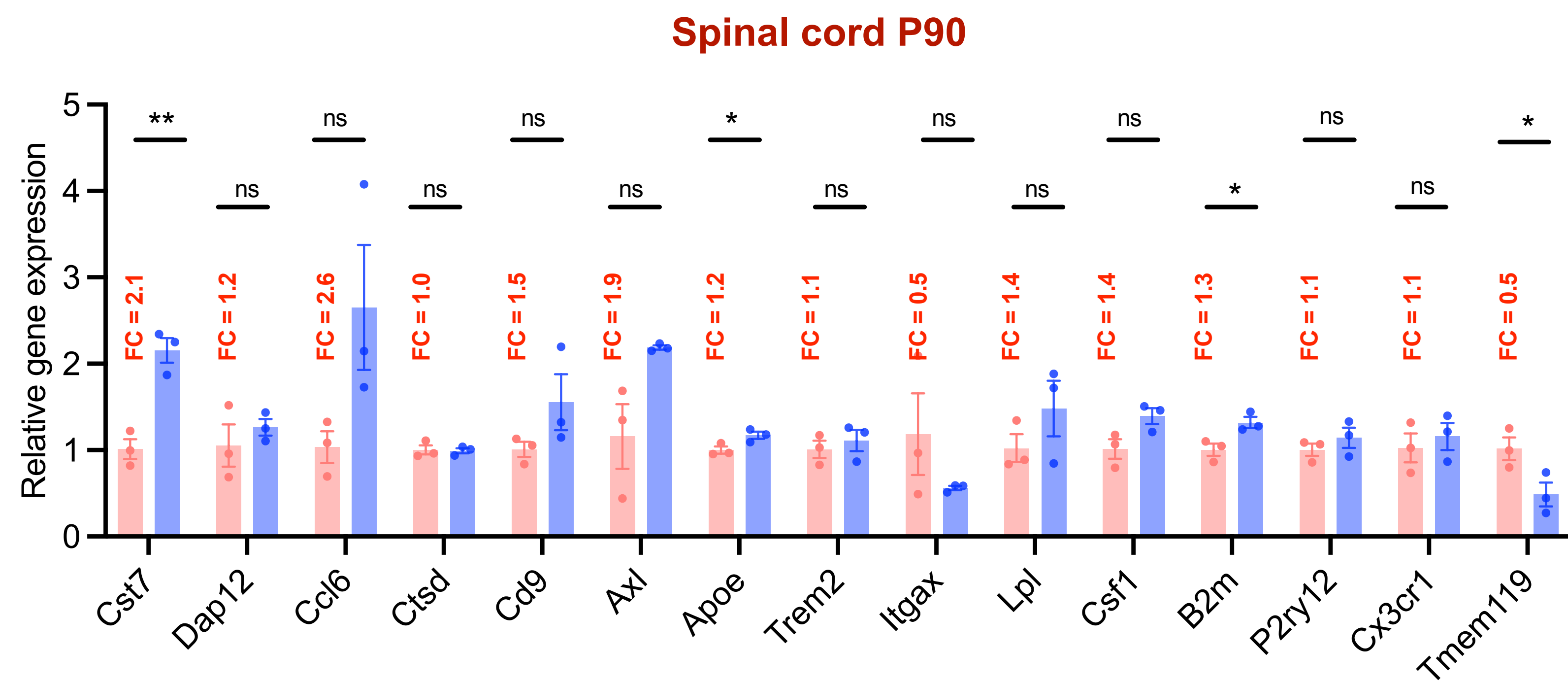

B

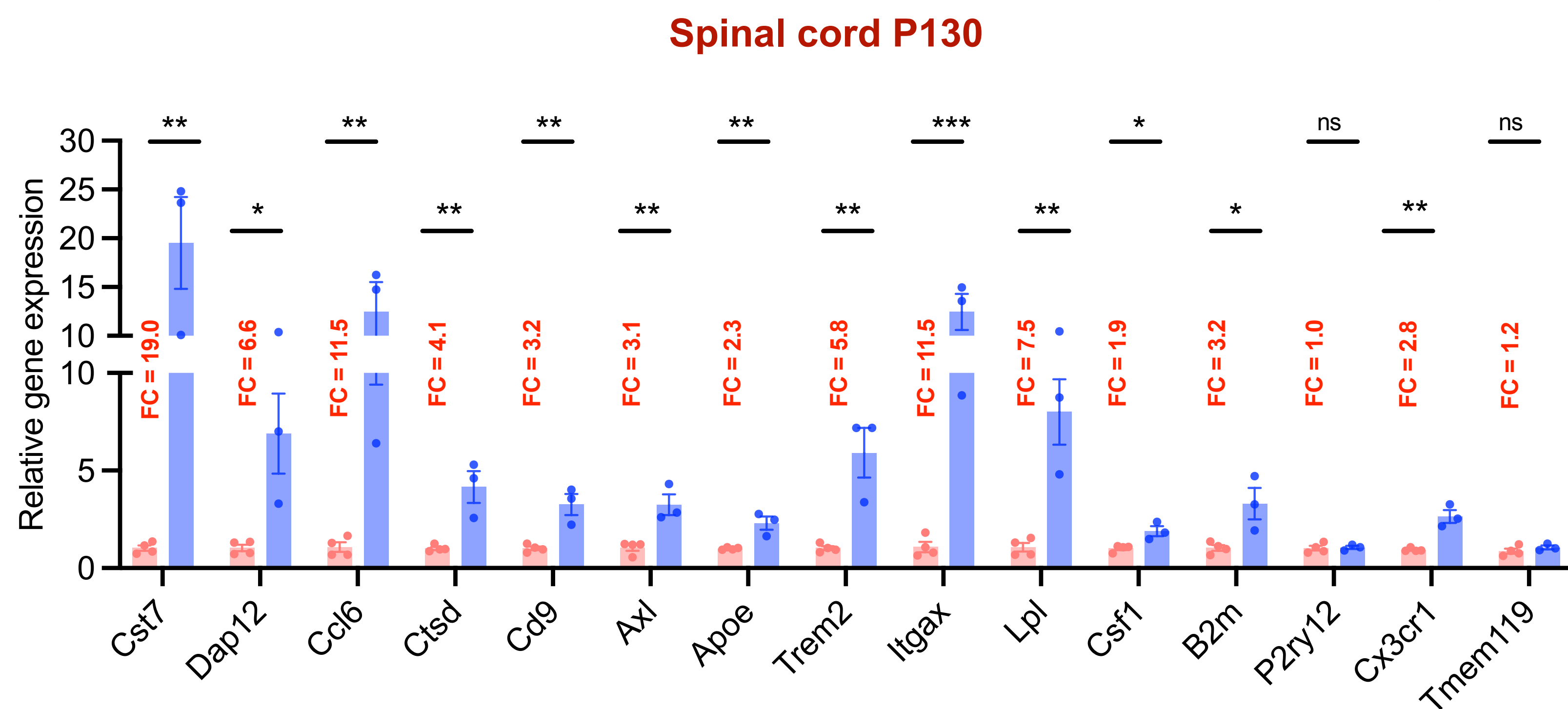

C

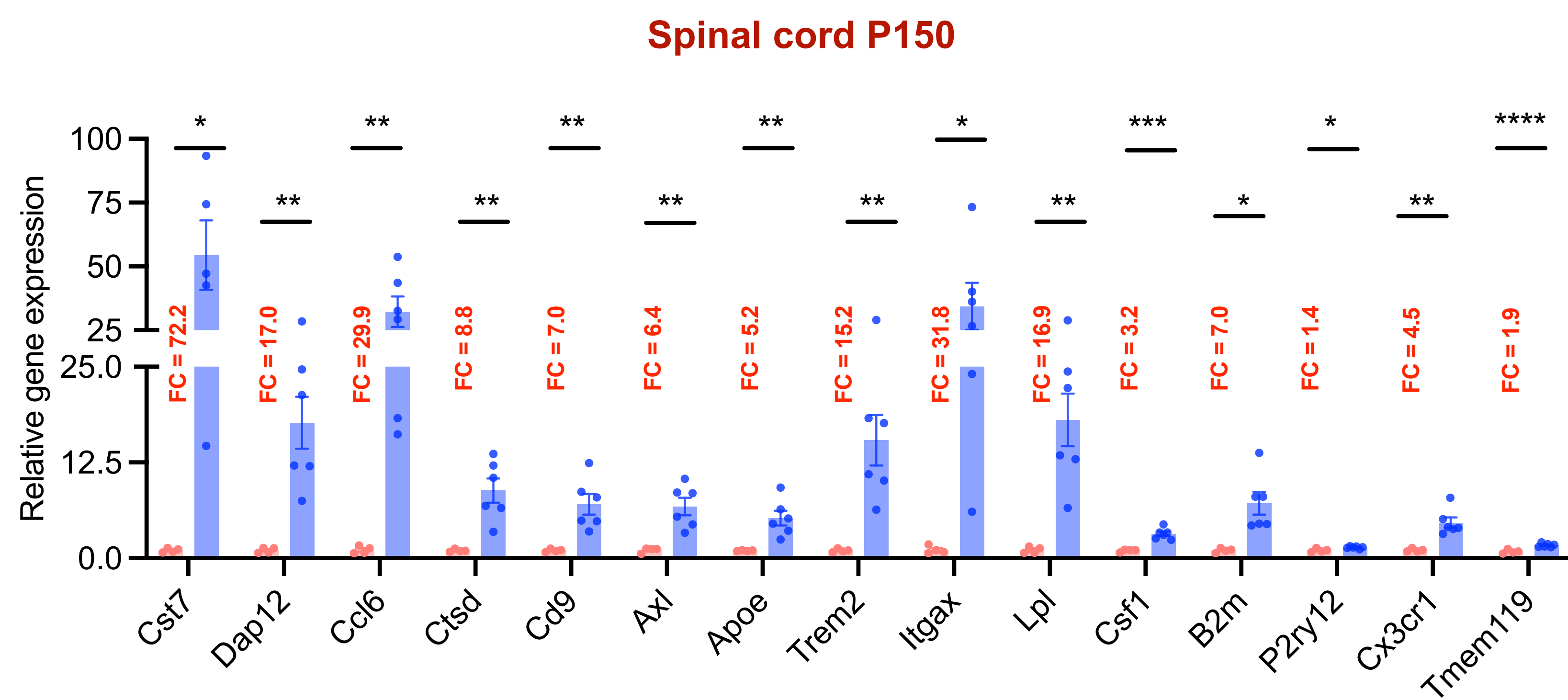

D

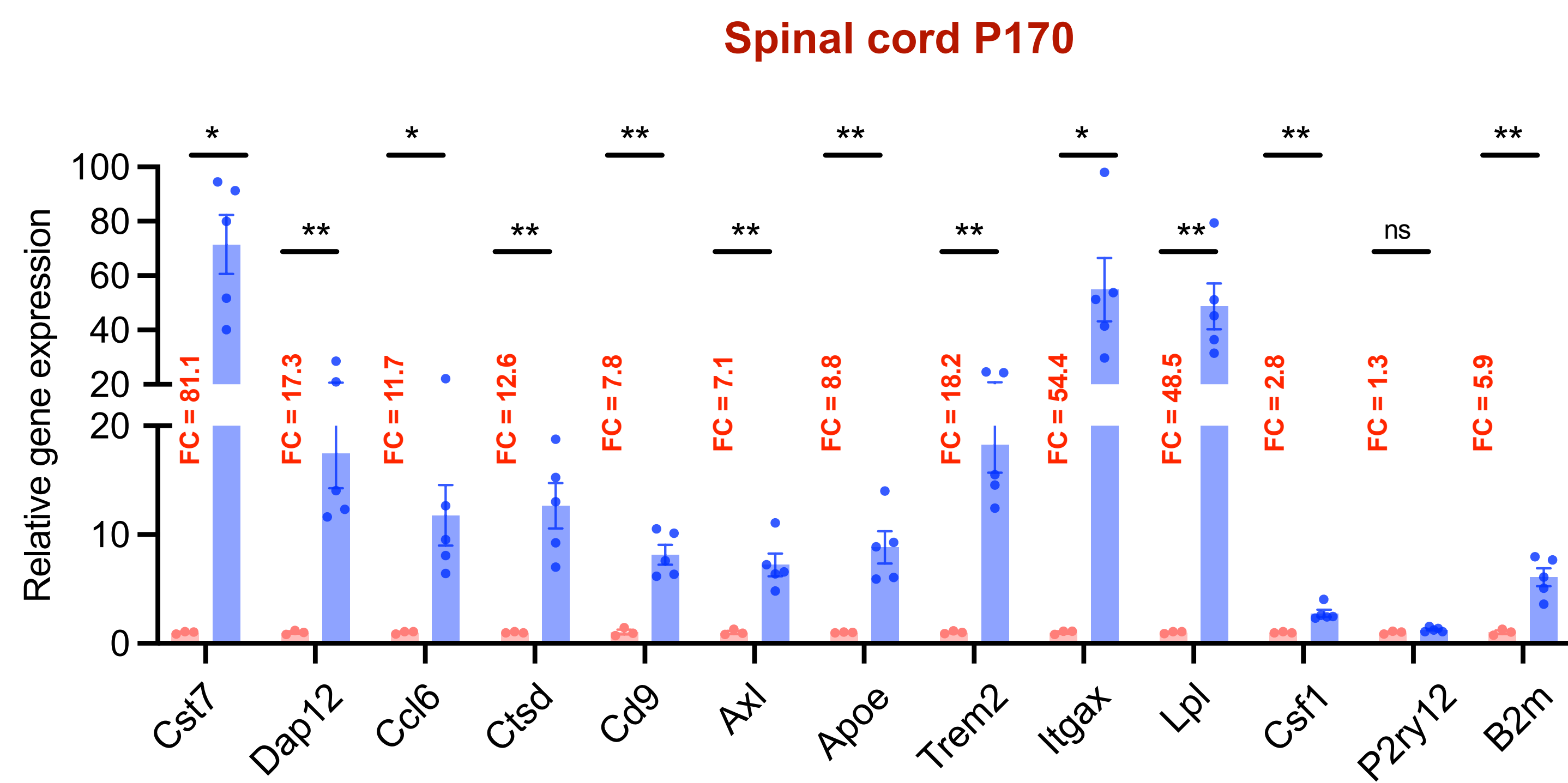

Supplement: Supplementary 1 — Figs. S1 to S9 Tables S1 to S14 [file research.0548.f1.zip › Figure S7.pdf]

A

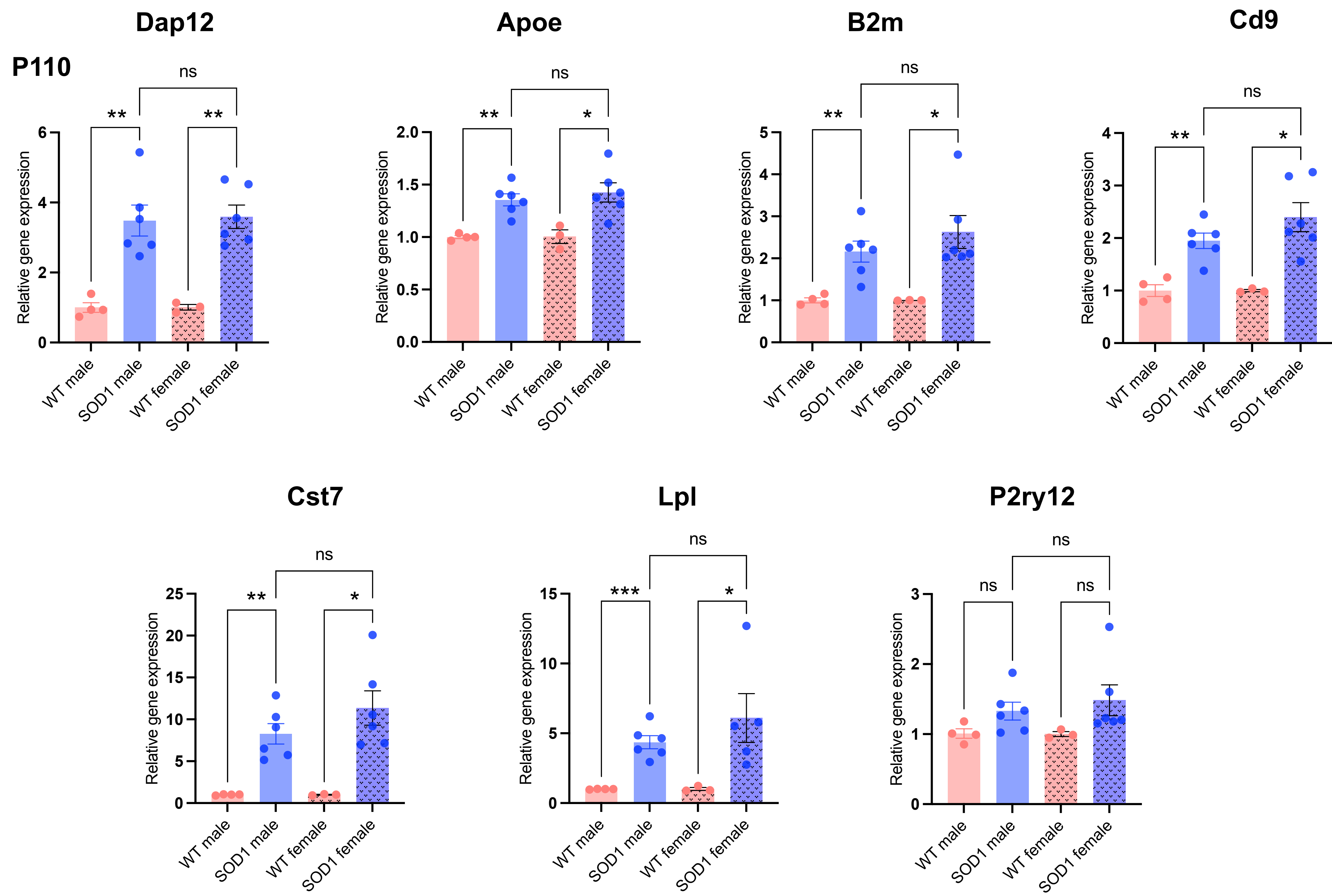

B

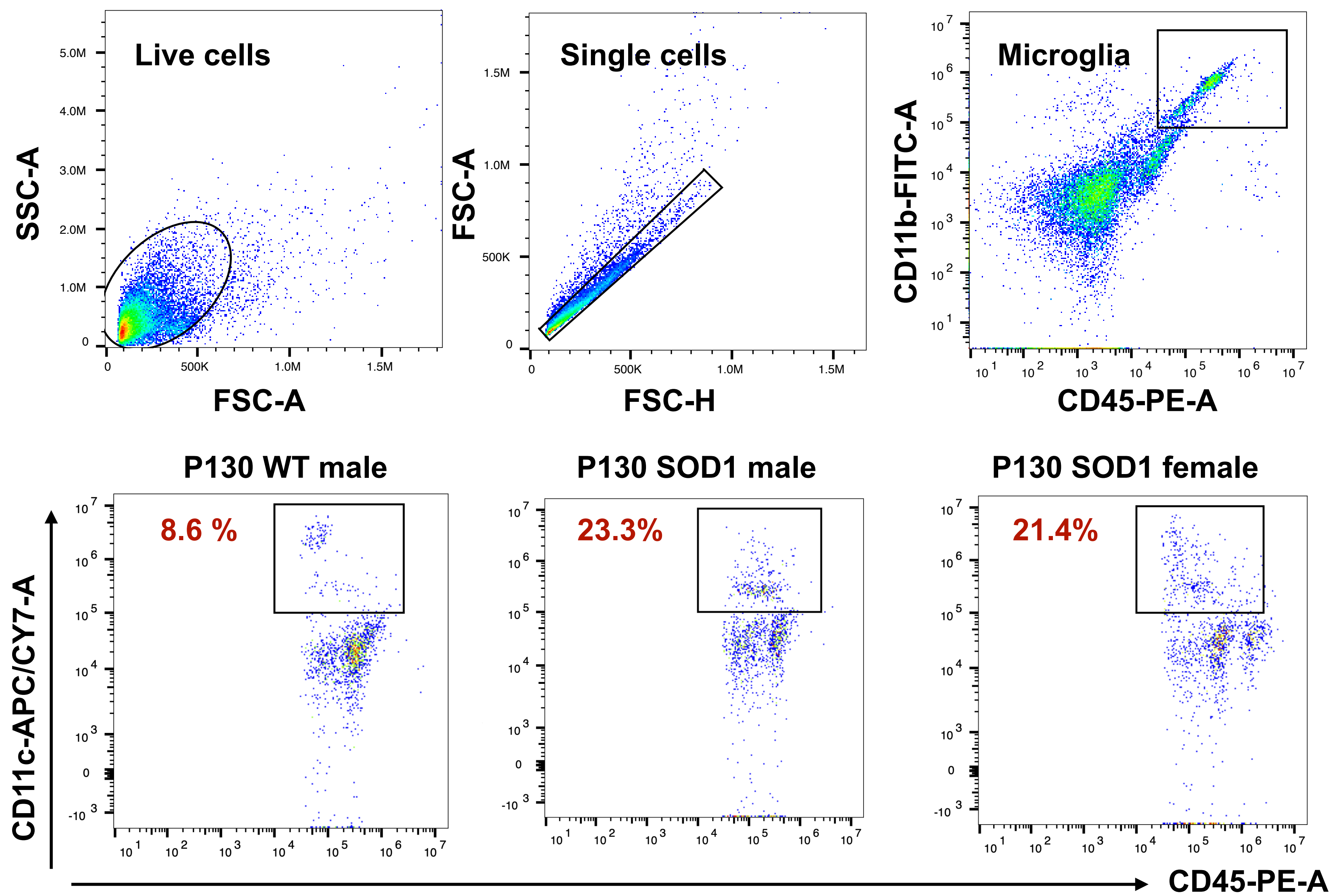

C

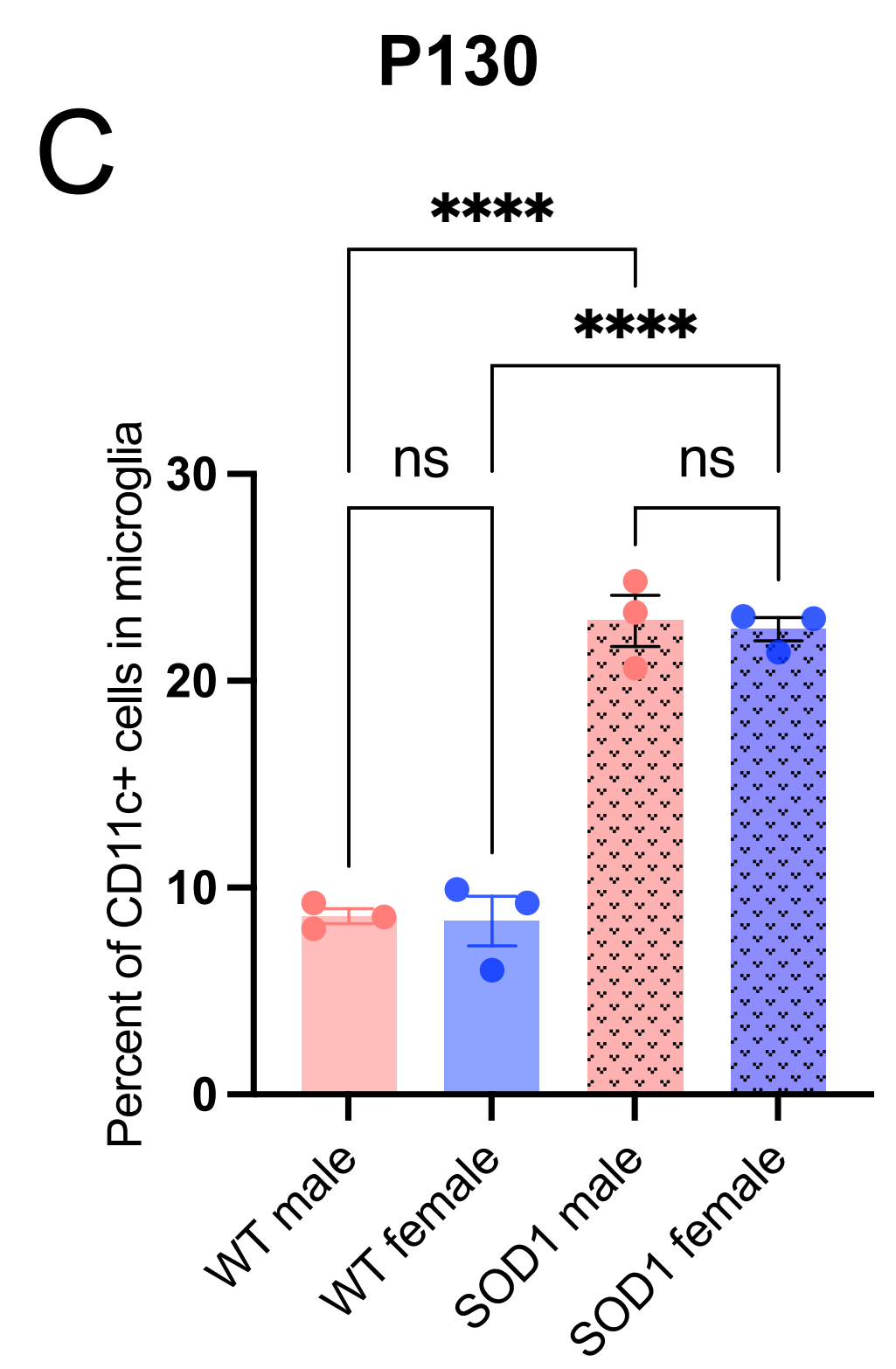

Supplement: Supplementary 1 — Figs. S1 to S9 Tables S1 to S14 [file research.0548.f1.zip › Figure S8.pdf]

A

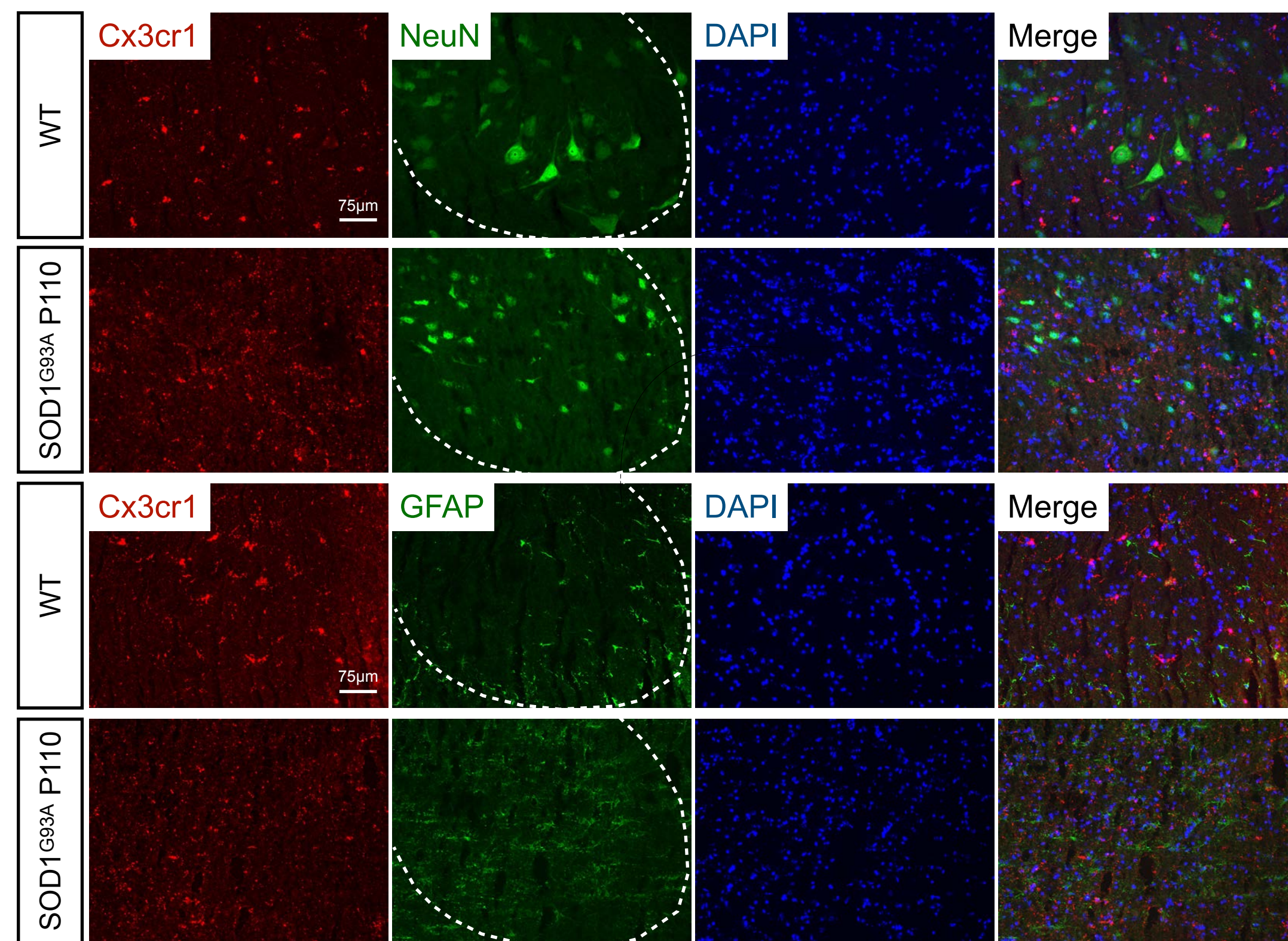

B

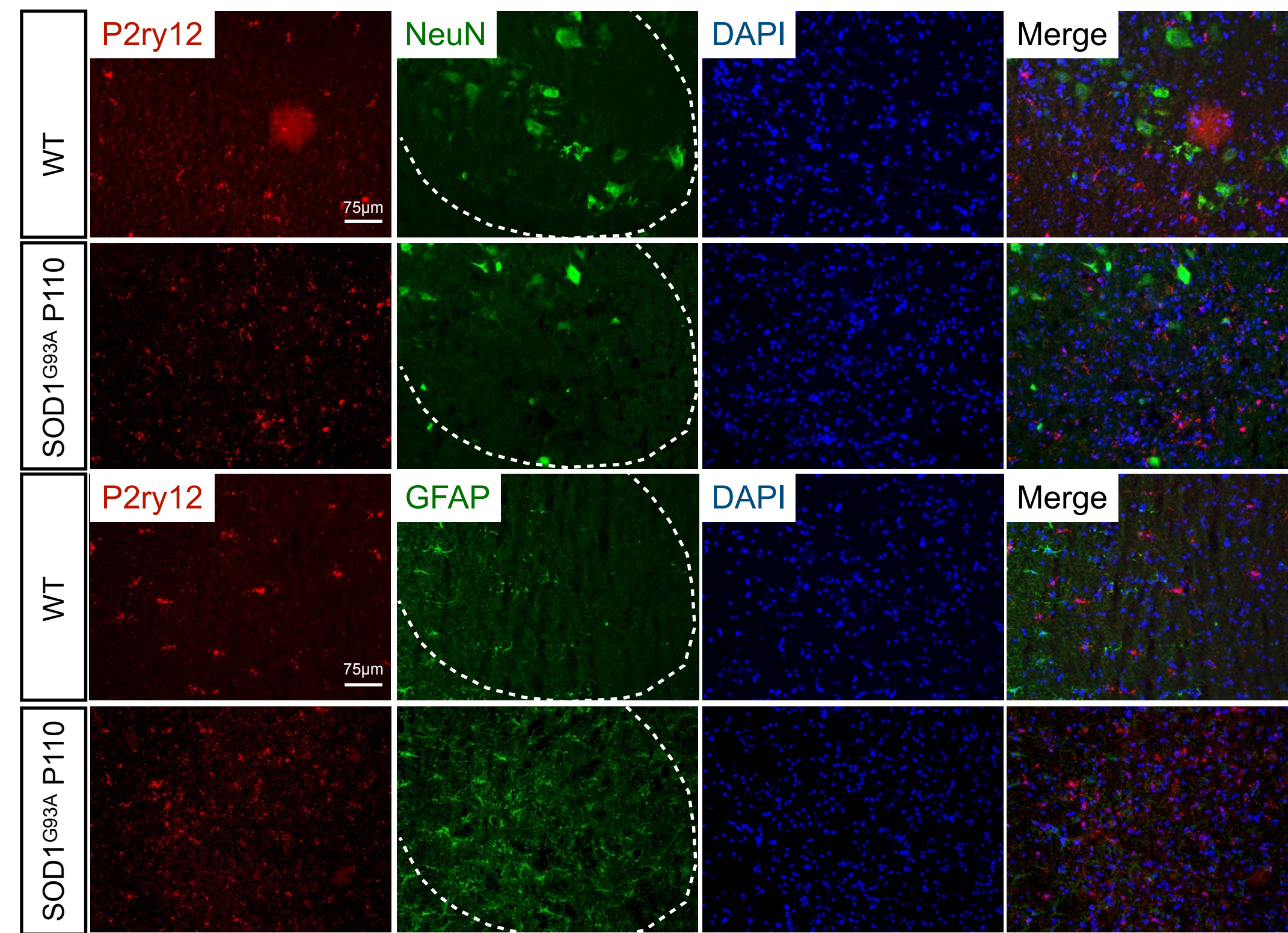

C

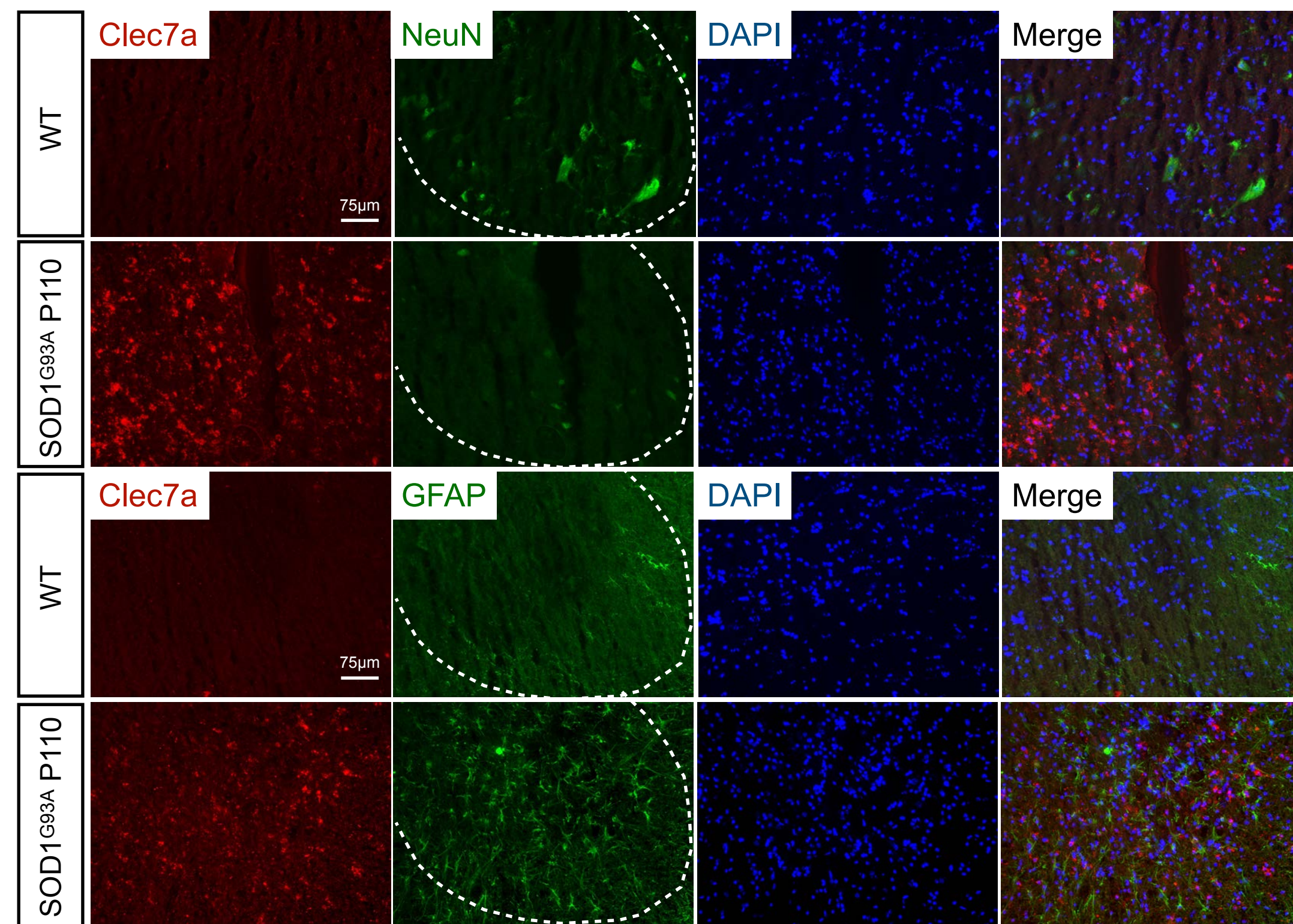

D

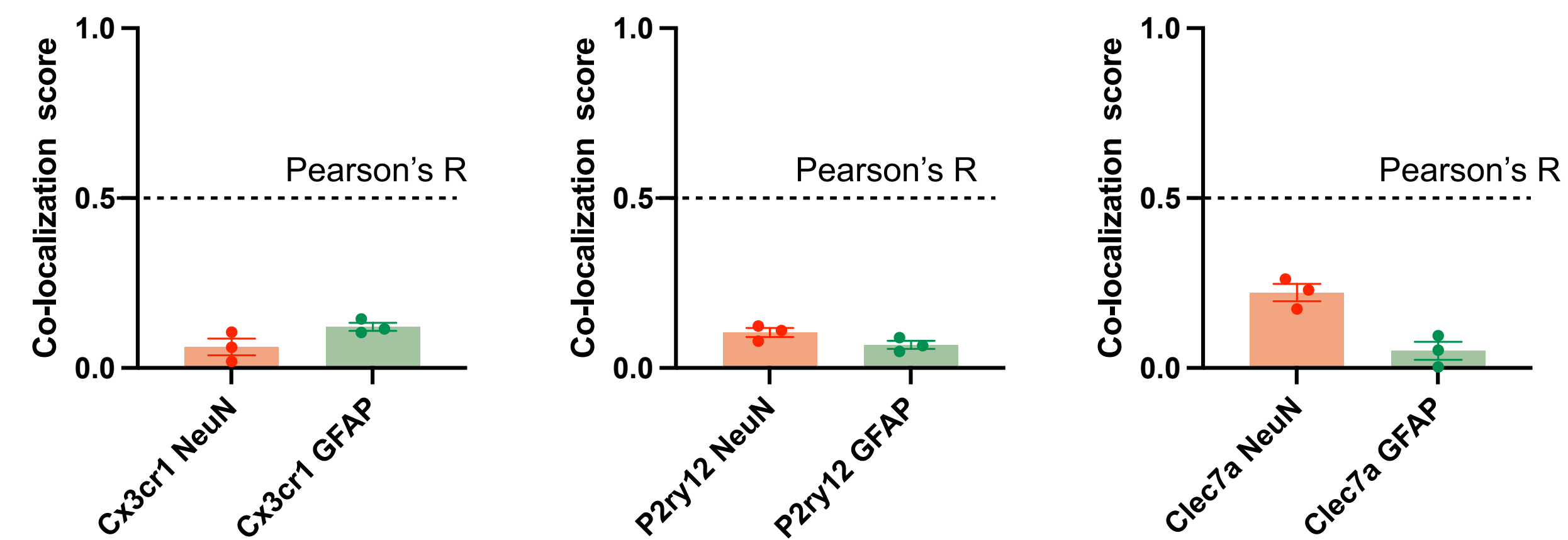

Supplement: Supplementary 1 — Figs. S1 to S9 Tables S1 to S14 [file research.0548.f1.zip › Figure S9.pdf]
